# Acinar micromechanics in health and lung injury: what we have learned from quantitative morphology

**DOI:** 10.3389/fphys.2023.1142221

**Published:** 2023-03-21

**Authors:** Lars Knudsen, Benjamin Hummel, Christoph Wrede, Richard Zimmermann, Carrie E. Perlman, Bradford J. Smith

**Affiliations:** ^1^ Institute of Functional and Applied Anatomy, Hannover Medical School, Hannover, Germany; ^2^ Biomedical Research in Endstage and Obstructive Lung Disease Hannover (BREATH), Member of the German Centre for Lung Research (DZL), Hannover, Germany; ^3^ Research Core Unit Electron Microscopy, Hannover Medical School, Hannover, Germany; ^4^ Department of Biomedical Engineering, Stevens Institute of Technology, Hoboken, NJ, United States; ^5^ Department of Bioengineering, College of Engineering Design and Computing, University of Colorado Denver | Anschutz Medical Campus, Aurora, CO, United States; ^6^ Department of Pediatric Pulmonary and Sleep Medicine, School of Medicine, University of Colorado Anschutz Medical Campus, Aurora, CO, United States

**Keywords:** micromechanics, pulmonary acinus, imaging, electron microscopy, stereology, Alveolar recruitment, interalveolar septa

## Abstract

Within the pulmonary acini ventilation and blood perfusion are brought together on a huge surface area separated by a very thin blood-gas barrier of tissue components to allow efficient gas exchange. During ventilation pulmonary acini are cyclically subjected to deformations which become manifest in changes of the dimensions of both alveolar and ductal airspaces as well as the interalveolar septa, composed of a dense capillary network and the delicate tissue layer forming the blood-gas barrier. These ventilation-related changes are referred to as micromechanics. In lung diseases, abnormalities in acinar micromechanics can be linked with injurious stresses and strains acting on the blood-gas barrier. The mechanisms by which interalveolar septa and the blood-gas barrier adapt to an increase in alveolar volume have been suggested to include unfolding, stretching, or changes in shape other than stretching and unfolding. Folding results in the formation of pleats in which alveolar epithelium is not exposed to air and parts of the blood-gas barrier are folded on each other. The opening of a collapsed alveolus (recruitment) can be considered as an extreme variant of septal wall unfolding. Alveolar recruitment can be detected with imaging techniques which achieve light microscopic resolution. Unfolding of pleats and stretching of the blood-gas barrier, however, require electron microscopic resolution to identify the basement membrane. While stretching results in an increase of the area of the basement membrane, unfolding of pleats and shape changes do not. Real time visualization of these processes, however, is currently not possible. In this review we provide an overview of septal wall micromechanics with focus on unfolding/folding as well as stretching. At the same time we provide a state-of-the-art design-based stereology methodology to quantify microarchitecture of alveoli and interalveolar septa based on different imaging techniques and design-based stereology.

## 1 An overview of the functional design of the lung

The design of the mammalian lung is optimized for efficient gas exchange, determined by micro- and ultrastructural properties of the fine lung parenchyma (Weibel et al., 1993). At the level of the gas-exchanger, the alveolus, blood and air are brought together over a large surface area and separated by a very thin leaflet of tissue that is referred to as the blood-gas barrier (Weibel and Knight, 1964; Gehr et al., 1978). At end-inspiration, more than 80% of the human lung is filled with air while blood within the pulmonary vasculature contributes roughly 10% to the lung volume. The remaining 10% of the lung is composed of tissue. In the gas-exchanging region, and above all the blood-gas barrier, the amount of tissue is reduced to a minimum to provide a short diffusion distance for gas exchange. The bulk of the connective tissue within the lung forms a sleeve around the purely conductive airway tree and the accompanying vessels, as well as in pleura and the connected interlobular septa (Knudsen and Ochs, 2018). These regions in which the bulk of connective tissue is located belong to the non-parenchyma of the lung (Ochs et al., 2004). The blood-gas barrier is a crucial part of the lung parenchyma and, in the human lung, has a surface area of 120–140 m^2^ and a harmonic mean thickness of less than 1 µm. Even though force bearing connective tissue elements are minimized in lung parenchyma, the large surface area is stable and maintains the precondition for effective gas-exchange during the respiratory cycle.

After entering the lung at the pulmonary hilum, the conductive airways of the human lung follow an irregular, dichotomous inside-out branching pattern. The last purely conducting airway is the terminal bronchiole. After an average of 14–16 branching generations the first airway with excrescent alveoli is termed transitional bronchiole (Haefeli-Bleuer and Weibel, 1988). Subsequent bronchioles with periodic alveoli are respiratory bronchioles. Once alveoli become maximally packed into a sleeve around the airway, the airway is termed an alveolar duct. Alveolar ducts resemble mesh-walled tubes formed of chicken wire. They do not have a continuous wall but are instead circumscribed by a boundary that is formed out of alveolar entrance rings.

Branches of the pulmonary arteries accompany the conductive airways constituting broncho-arterial units (Weibel and Gomez, 1962). The smallest lung unit that is in part bounded by connective tissue is the (secondary) pulmonary lobule (Webb, 2006). Paired bronchioles/arteries enter the center of the lobule; veins and lymphatic vessels are located in the connective tissue of the interlobular septa. The acinus, the functional unit of the gas-exchanging region, starts with a transitional bronchiole (Haefeli-Bleuer and Weibel, 1988). Human, rat, and mouse lungs contain approximately 30,000, 6,000 and 600 acini, respectively (Haefeli-Bleuer and Weibel, 1988; Vasilescu et al., 2012; Barré et al., 2014; Barré et al., 2016). Within the human acinus, the first generations of airways are respiratory bronchioles. The airways continue the dichotomous branching pattern for, on average, eight generation. Within the rodent acinus, in contrast, the airways are nearly all alveolar ducts. Within the acinus, the alveoli are blind-ended while the vessels enable through-flow (König et al., 1993).

The essential consequences of the dichotomous branching pattern of the airways are twofold: first, the airway tree is space filling within the thorax so that air (and blood) can reach every part of the lung efficiently. Secondly, the number of airways increases by a power of two per branching generation which is accompanied by an exponential increase in the cumulative airway cross-sectional area in subsequent airway generations (Weibel and Gomez, 1962). Consequently, the airflow velocity decreases dramatically from the central to the peripheral airways. Gas transport is purely convective from the airway opening—the nose during spontaneous breathing or the trachea during mechanical ventilation—to the terminal bronchiole. There is a combination of both convective and passive diffusive gas transport within the acinar airways (Sapoval et al., 2002; Hogg et al., 2017). During quiet spontaneous breathing, the transport of gases is primarily by convection within the first three branching generations of intra-acinar airways. In the more peripheral branching generations of the acinus, diffusion dominates convection in terms of gas transport. The part of the pulmonary acinus in which diffusion dominates convection has been termed a diffusion cell. A diffusion cell has approximately the size of one-eighth of the acinus during quiet spontaneous breathing in humans (Sapoval et al., 2002). Increasing the tidal volume during exercise or sighing, shifts the location of transition from convection-dominated to diffusion-dominated gas transport to more peripheral generations of the intra-acinar airways.

The pulmonary acini form the fine lung parenchyma. In the human lung, the parenchyma contains an average of 480 million alveoli and contributes roughly 90% to total lung volume. The remaining 10% of the volume is assigned to structures which do not contribute directly to gas exchange and includes purely conductive airways, larger blood-vessels, and the majority of connective tissue which embeds these conductive airways and vessels (Ochs et al., 2004). Alveoli are separated from each other by interalveolar septa and three-dimensional imaging studies have shown that each alveolus in the human lung has 7–9 neighboring alveoli (Reimelt et al., 2023). The interalveolar septa contain a dense network of capillaries composed of capillary segments whose lengths are in the range of their diameter. Hence, the morphology of the alveolar capillary network has been described as sheet-flow around pillars that reach from one side of the interalveolar septa to the other and are filled with cells—principally fibroblasts but also alveolar epithelial cell bodies. Thus, the morphology of the alveolar capillary network differs markedly from that of capillary networks found in the systemic circulation (Fung and Sobin, 1969; Mühlfeld et al., 2010; Grothausmann et al., 2017; Buchacker et al., 2019).

**FIGURE 1:**
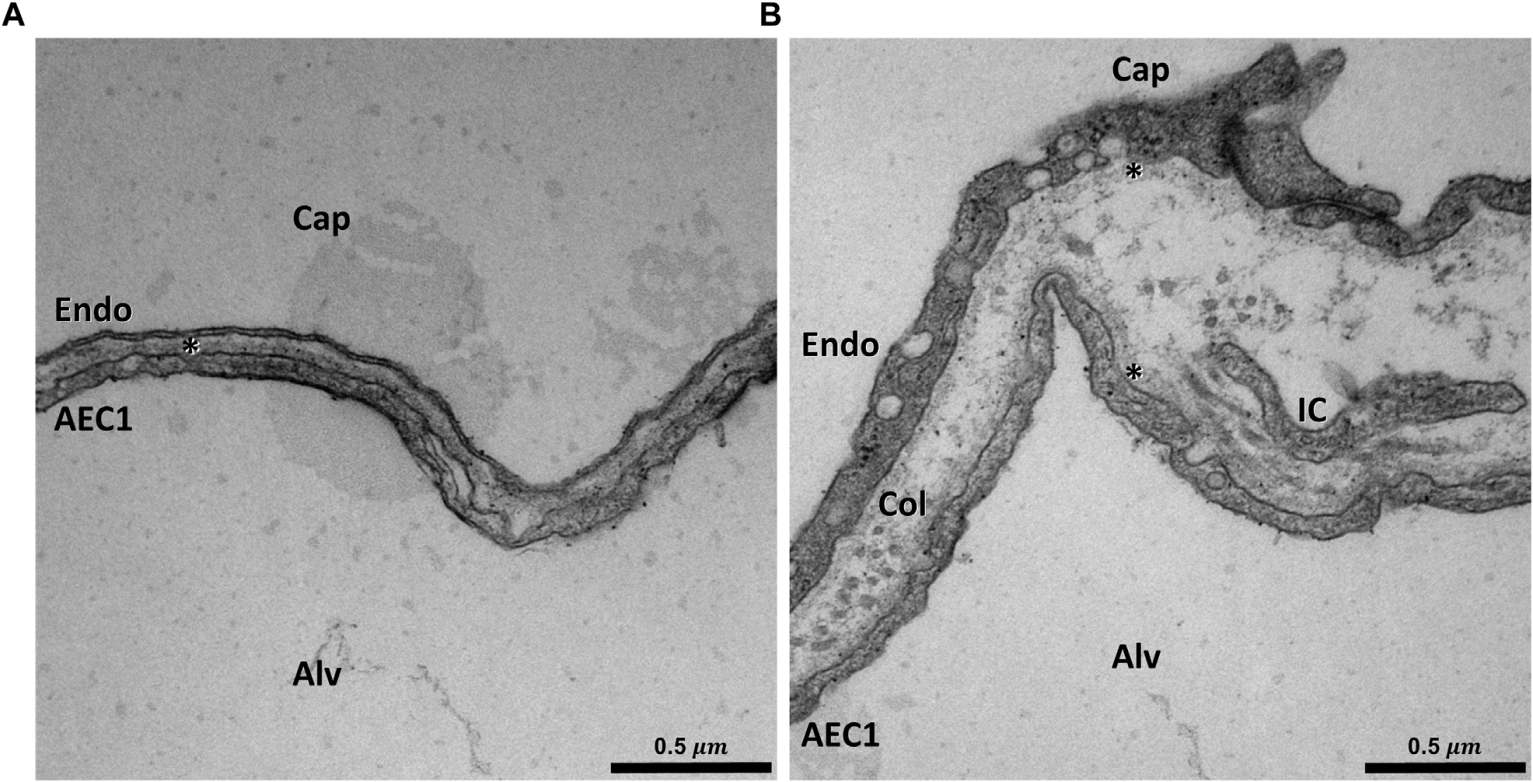
Ultrastructure of the blood-gas barrier: Transmission electron micrograph of a rat lung fixed *in vivo* by vascular perfusion *via* the vena cava caudalis at an airway opening pressure of 5 cmH_2_O on expiration after two recruitment maneuvers (3 s pause at 30 cmH_2_O) (Knudsen et al., 2018). The capillaries (Cap) are open and free from blood cells. In **(A)** an example of the thin part of the blood-gas barrier can be seen. In this area the squamous extension of an alveolar epithelial type 1 cell (ACE1), the basement membrane (asterisk) and the endothelial cell (Endo) form the blood-gas barrier. In **(B)** an example of the thick part of the blood-gas barrier is illustrated. The interstitium between the AEC1 and the endothelium is widened and both AEC1 and the endothelial cell have a basement membrane (asterisk) of its own. Aside from collagen fibrils (Col), cell extensions of interstitial cells (IC), e.g. fibroblasts can be identified.

The capillary lumen is separated from the alveolar airspace by the blood-gas barrier. The blood-gas barrier consists of the alveolar epithelium, the interstitial space, and the endothelium and forms a continuous boundary around the air in an alveolus that is interrupted only by the alveolar entrance and by inter-alveolar pores of Kohn. Approximately 50% of the blood-gas barrier of the human lung is termed the “thin side” of the capillary network. Here, the interstitial space contains only a single basement membrane (BM), which is shared by both the alveolar epithelium and the capillary endothelium (Weibel, 2017). The other half is the “thick side” on which the BM of the epithelium is separated from that of the endothelium so that the widened interstitium provides space for stabilizing fiber elements, e.g., collagen fibrils and elastic fibers, and also extensions of interstitial cells such as fibroblasts. The thick side contains elements of a very economically constructed fiber system and other components of the extracellular matrix (Figure 1). This fiber system determines the micromechanical properties of the interalveolar septa (Bou Jawde et al., 2020) and contributes to mechanical stabilization of the gas-exchanging surface area. In addition, the thick side accommodates excess interstitial fluid, thus preserving the gas exchange function of the thin side (Weibel, 2009; Beretta et al., 2021; Mostaco-Guidolin et al., 2021).

**FIGURE 2:**
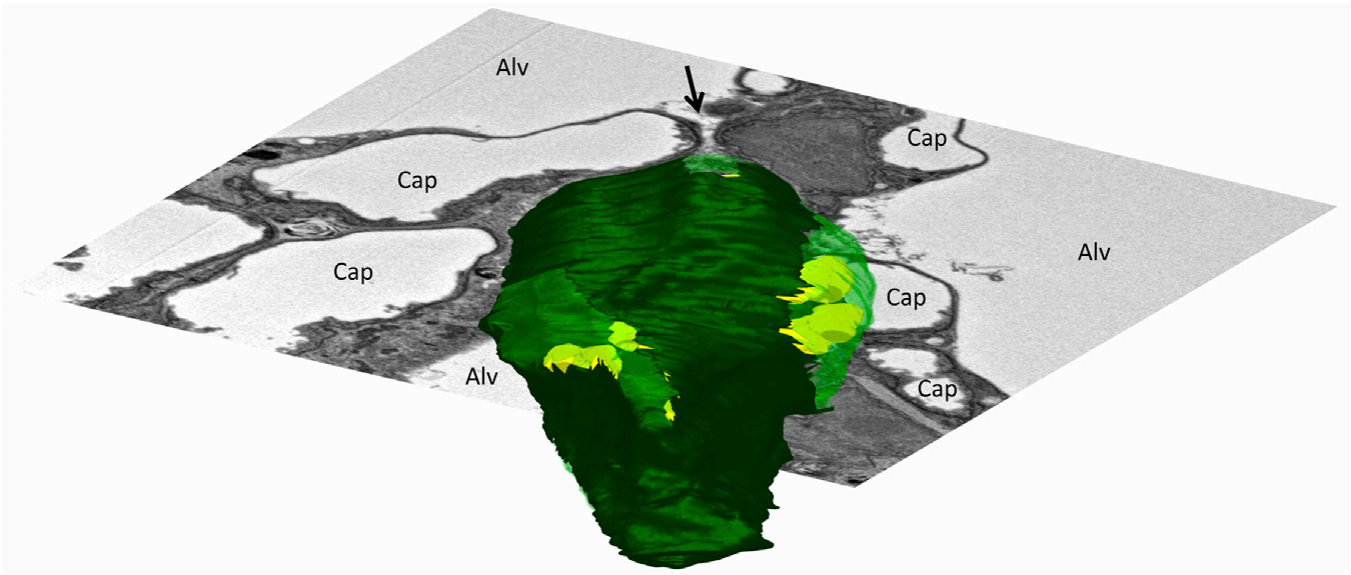
Three-dimensional model of an alveolar epithelial type 2 (AE2) cell: The mouse lung was fixed *in situ* by vascular perfusion *via* the right ventricle at an airway opening pressure of 2 cmH_2_O on expiration after two recruitment maneuvers (3 s pause at 30 cmH_2_O) (Ruhl et al., 2019). Tissue was processed for serial block face scanning electron microscopy (SBF-SEM) as described in Buchacker et al., 2019. The EM image stack was used for segmentation of an AE2 cell, located within a junction of three interalveolar septa. On a three-dimensional representation of the AE2 cell surface, apical portions of the plasma membrane are shown in light green and basolateral portions that are attached to the epithelial basement membrane are shown in dark green. Lamellar bodies located below the plasma membrane are shown in yellow. The AE2 cell is, further, shown inserted into a two-dimensional electron microscopic image of the surrounding environment. The AE2 cell shows multipolarity with four apical domains being in contact with three alveolar airspaces (Alv). The arrow points into a pleat filled with protein containing fluid (hypophase). The bottom of the pleat is formed in part by one of the apical domains of the AE2 cell.

The alveolar epithelium contains 2 cell populations in a healthy lung, namely the squamous alveolar epithelial type 1 (AE1) and the more cube-shaped alveolar epithelial type 2 (AE2) cells. Based on single cell RNA-sequencing, a third population, termed alveolar epithelial type 0 (AE0) cell has recently been described in the human lung in those interalveolar septa that are attached to the respiratory bronchioles. This morphologically not yet well characterized cell population has been suggested to fulfill functions in lung regeneration (Kadur Lakshminarasimha Murthy et al., 2022). The AE1 cells minimize the thickness of the blood-gas barrier. AE1 cells cover the capillary network on the air side with planar cellular extensions attached to the underlying BM. The bodies of the AE1 cells, which contain the nucleus and most of the organelles, are usually located in the pillars between the meshwork of the alveolar capillary network. These pillars allow the AE1 cells to traverse the interalveolar septum and form further planar cellular extensions on the other side of the interalveolar septum so that an AE1 cell usually faces more than one alveolar airspace (Schneider et al., 2019). The population of AE1 cells covers approximately 95% of the epithelial BM in both human (Gehr et al., 1978) and mouse lungs (Ruhl et al., 2019; Engelmann et al., 2021). The remaining 5% of the epithelial BM is covered by the cube-shaped, secretory AE2 cells, which are twice as numerous as AE1 cells (Crapo et al., 1982). The AE2 cells synthesize, store, and secrete pulmonary surfactant into the alveolar airspace, where the surfactant dynamically reduces surface tension of the alveolar liquid lining layer. In addition, AE2 cells have stem cell properties. They have the potential for self-renewal and are able to transdifferentiate into AE1 cells so that AE2 cells are responsible for the regeneration of the alveolar epithelium under physiological and pathophysiological conditions (Barkauskas et al., 2013; Jansing et al., 2017). The AE2 cells are usually located in the corners of the alveoli or in niches between segments of the alveolar capillary network, and thus tend not to interfere with the gas-exchanging function. Like the AE1 cells, AE2 cells also traverse the interalveolar septa and are part of the pillars located between the segments of the alveolar capillary network. Thereby, AE2 cells are in direct contact with more than one alveolus (Figure 2). This configuration strategically positions one AE2 cell to be able to supply surfactant to and regenerate the alveolar epithelium in multiple alveoli *via* its multipolar apical domains (Konkimalla et al., 2022).

## 2 Stabilization of the pulmonary acinus: Balance between distending inflation pressure, retracting fiber elasticity, and surface tension

The sole distending stress applied to the lungs is the transpulmonary pressure, which can be separated into the resistive pressure drop along the airways and the pressure across the pleural surface (Mead et al., 1970). The latter equals alveolar pressure minus pleural pressure and is known as the elastic recoil pressure because it is countered by inwardly-directed stresses due to fiber elasticity and interfacial surface tension. The fiber network of the lungs comprises three distinct systems (Wagner et al., 2015; Weibel, 2017; Mostaco-Guidolin et al., 2021). Located most centrally is the *axial system*, which enters the lung at the pulmonary hilum and follows the run of the dichotomous branching of the conducting airways. It reaches the acinus with the terminal bronchiole and ends with fibers forming a lattice around the alveolar entrances, thereby providing a boundary for the alveolar duct. In essence, the axial system enters the acinus centrally and wraps the intra-acinar airways, particularly the alveolar ducts. Located most distally is the *peripheral system*, which originates from the pleura and continues into the interlobular septa. Located in between, and linking, the axial and peripheral systems is the delicate *septal fiber system*, which occurs in the thick parts of the blood-gas barrier and interlaces the capillary network. It is less stiff than either the axial or the peripheral fiber system. Thus, the three fiber systems are arranged roughly in series. Surface tension acts along the alveolar septa, thus in parallel with the septal fiber system. Overall, the balance between distending inflation pressure and retracting fiber elasticity and surface tension comprises a self-stabilizing tensegrity structure (Ingber, 2003).

The effect of surface forces on the lung microarchitecture was elucidated with the development of appropriate tissue processing for electron microscopic investigation (Weibel and Gil, 1968; Untersee et al., 1971). Studies employing these techniques revealed that the alveolar space is not dry. Instead, the alveolar epithelium is covered by a thin liquid lining layer. The liquid layer is aqueous with some protein content, harbors macrophages and is coated by a surface layer of phospholipids (Weibel and Gil, 1968; Reifenrath and Zimmermann, 1973). Quantitative electron microscopic investigations of lung parenchyma from rats following chemical-free cryo-fixation demonstrated that the thickness of this liquid lining layer (= hypophase) varies dramatically (Bastacky et al., 1995). On top of the planar, squamous extensions of the AE1 cells the average thickness of the hypophase is 140 nm and the air-liquid interface is close to planar. In the corners of the alveoli, where alveolar walls converge and AE2 cells are located, the hypophase has a height of up to 890 nm and the interface is curved. With the presence of an air-liquid interface, surface tension forces must act in the lungs. The magnitude of lung surface tension, however, is reduced by the presence of the pulmonary surfactant.

Pulmonary surfactant is a mixture of phospholipids, neutral lipids and surfactant-associated proteins. The most essential components of surfactant for reducing surface tension are palmitoylated phospholipids and surfactant proteins B and C (Notter et al., 2002; Schürch et al., 2010; Knudsen et al., 2012; Ruwisch et al., 2020). Most, but not all components of the pulmonary surfactant are stored in specialized, lysosome-derived organelles, the lamellar bodies (Schmiedl et al., 2005; Ochs, 2010). Lamellar bodies are sheathed by a limiting membrane and contain very densely packed biomembranes with an onionskin-like morphology. Upon stimulus, e.g., stretch during a sigh, the limiting membrane fuses with the apical plasma membrane of the AE2 cell and the surfactant is released into the alveolar space (Wirtz and Dobbs, 1990). Here, the content of the lamellar body unfurls and forms a network of membranes termed tubular myelin, characterized by piles of parallel-organized membranes connected by intersecting membrane planes (Lettau et al., 2022). Tubular myelin or, alternatively, sub-interfacial stacks of surfactant bilayers form reservoirs of active surfactant within the alveolar space (Schürch et al., 2001; Bachofen et al., 2005; Dietl and Haller, 2005). The reduction in surface tension that surfactant achieves is dramatic, particularly with near-maximal interfacial surfactant density at end-expiration. With increasing lung volume towards total lung capacity (TLC) surface tension increases due to reduced interfacial surfactant density, but that density is still high. At about 80% TLC, interfacial surfactant is still in the liquid-condensed state, such that surface tension is still relatively low (Schürch et al., 1976; Nag et al., 1998; Nguyen and Perlman, 2020). Alveolar surface tension cannot, presently, be determined *in vivo* but has been determined *in situ* in excised lungs by two micropuncture-based methods—one employing deposition of surface tension-sensitive liquid droplets and one combining servo-nulling measurement of alveolar liquid phase pressure, confocal microscopic determination of interfacial radius, and application of the Laplace relation (Schürch, 1982; Kharge et al., 2014). At a given lung inflation pressure, these methods showed that surface tension is the same between different-sized alveoli and between aerated and flooded alveoli. Neither method is capable of revealing if surface tension varies between the planar interface, along most of the alveolar septum, and the curved interface in the alveolar corners. Regardless of whether there is intra-alveolar variation in surface tension, however, the intra-alveolar variation in interfacial curvature has profound effects.

**FIGURE 3:**
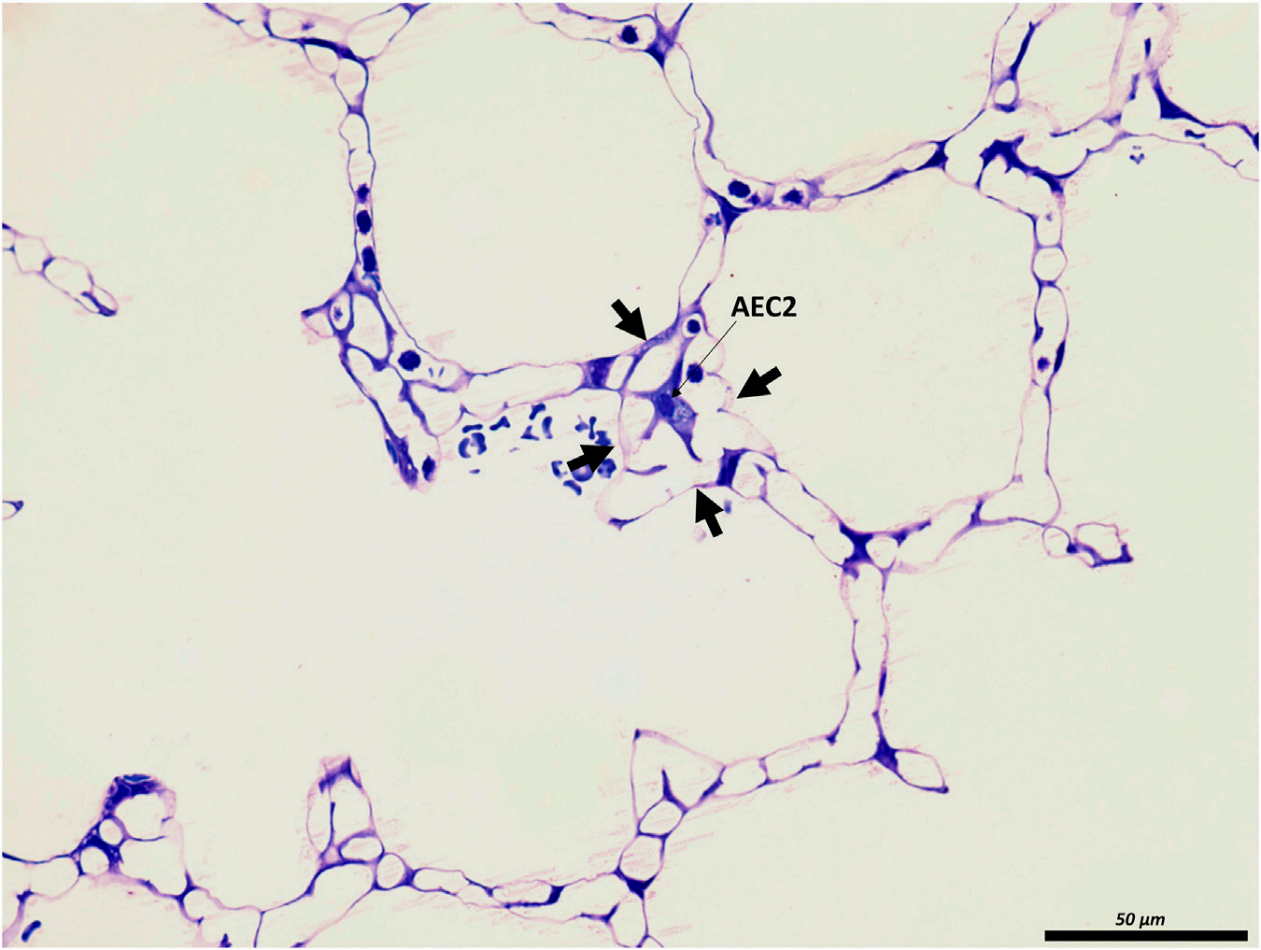
The junction of inter-alveolar septa: A healthy rat lung was fixed *in vivo* by vascular perfusion *via* the vena cava caudalis at an airway opening pressure of 10 cmH_2_O on expiration after two recruitment maneuvers (3 s pause at 30 cmH_2_O) (Knudsen et al., 2018). In the middle of the septal junction (arrows), an alveolar epithelial type II cell (AEC2) is present. While most of the interalveolar septa contain a single layer of the alveolar capillary network, there appear to be two layers at this junction surrounding the AEC2, most likely due to pleating that causes piling up of the interalveolar septa.

The physiological effects of surface tension on the microarchitecture of lung parenchyma were first seen more than 40 years ago by Gil and coworkers. Comparison of air- and saline-filled lungs fixed by vascular perfusion at the same lung volumes (given as percentage of TLC) revealed dramatic differences in the microscopic appearance of the acinar microarchitecture (Gil et al., 1979). The pressures needed to achieve a defined lung volume were much lower in the saline compared to air-filled lungs. At low volume in saline-filled lungs, in the absence of surface tension, septal capillaries bulge into the lumen such that septa have an uneven surface area and markedly varied thickness along their length. The central splines of many septa follow an undulating, unconstrained, path. At high volume above 80% TLC, the capillaries remain bulging but forces transmitted through the septa tend to straighten their central splines. In the normal air-filled lung surface tension is present and the tendency of surface tension is to minimize interfacial surface area. Even the low surface tension present at low lung volume has a marked effect. Surface tension reduces interfacial surface area by flattening capillaries and, through imposition of septal pleating, reducing septal undulation. Septal pleats are small folds in the alveolar septa where the adjacent epithelial surfaces are in contact with each other instead of exposed to air and are detailed in section 3.3, below. But surface tension effects vary along the lengths of the septa. Along most of the septal length, where the air-liquid interface is planar, there is not, according to the Laplace relation, any pressure drop across the interface. With full pulmonary inflation (100% TLC), capillaries are flattened and no pleats are present; there is a single layer of capillaries and the septal spline is linear, suggesting it is in tension. In the alveolar corners, the air-liquid interface is curved such that, according to Laplace, there is a pressure drop across the interface and liquid pressure is less than air pressure. With a lung inflation pressure of 15 cmH_2_O, corner liquid pressure was measured and found to be a mere 2 cmH_2_O (Kharge et al., 2014). With reduced liquid pressure acting on the septal tissue, capillaries remain patent. Further, the lower liquid pressure in the alveolar corners may draw septal tissue toward the corners and be responsible for septal pleating that is observed at septal junctions. Consequently, in the alveolar corners, there are piles of well-perfused capillaries (Figure 3). At high lung volumes, along most of the septal length, capillaries are further flattened into a slit-like morphology but at the alveolar corners capillaries are still patent. Also, with increasing lung volume there is progressive unfolding of septal pleats and, as detailed below, the septal fibers bear more stress. Overall, the effect of surface tension is to straighten the septal spline; compress the septal tissue—albeit less so near alveolar corners such that septal thickness tends to increase toward septal endpoints; and smooth the septal surface. The liquid lining layer, which fills crevices and has a variable height along the full lengths of the septa (Bastacky et al., 1995), also contributes to the smoothing of the septal surface. The distribution and the mean thickness of the hypophase appear to be subject to dynamic changes during breathing and depend on the lung volume (Ruhl et al., 2019). Since the hypophase, including intra-alveolar surfactant, plays a central role with regard to the pulmonary microarchitecture, studies designed to investigate aspects of the acinar micromechanics need to be performed in air-filled lungs under well controlled conditions, e.g., pressures at the airway opening, lung volume history, and pulmonary vascular pressure.

It is well accepted that low surface tension at low lung volume prevents destabilization of the gas-exchanging surface area (Bachofen and Schürch, 2001). Although an alternative analysis suggests that low surface tension may not be a prerequisite for end-expiratory lung stability (Reifenrath, 1975), the importance of low surface tension is supported by the observation that increased surface tension destabilizes the gas-exchanging surface area, particularly at low lung volumes where the alveoli are small and interfacial radius is low (Mercer et al., 1987; Knudsen et al., 2018; Smith et al., 2020). Destabilization could be attributable to the curved interface in the alveolar corner. According to the Laplace relation, a smaller than normal interfacial radius in the corner of the alveolus would lead to a lower than normal liquid pressure in the corner which should cause more septal folding than normal in the corner. One could imagine that as septa crumple more than usual at their ends, septal length would decrease, the low corner liquid pressure would be translated toward the centers of the septa and positive feedback could lead to alveolar collapse.

At the acinar level, an important effect of lung inflation is to increase alveolar duct volume relative to alveolar volume. Consequently, inflation increases alveolar surface area less than it would if alveolar volume increased proportionately with lung volume (Bachofen et al., 1987; Mercer et al., 1987). Based on the organization of the lung fiber system and the reported morphological features of air- and saline-filled lungs (Gil and Weibel, 1972; Gil et al., 1979; Bachofen et al., 1987), Wilson and Bachofen created a much-appreciated model that explained the mechanical interdependence between alveolar duct and alveoli (Wilson and Bachofen, 1982). The model is valid for lung volumes up to 80% of TLC, which include the physiological range of spontaneous breathing (Wilson and Bachofen, 1982). Recalling that the three lung fiber systems are arranged roughly in series, they can be thought of as three springs connected in series. Applying an inflation pressure to the lung is analogous to applying a constant tension to the three springs, and causes greatest distension of the least-stiff spring. Thus, in healthy lungs at low volume, the stiff axial fibers keep the duct relatively small while, due to distension of compliant septal fibers, the alveoli are stretched relatively large. But the septal fiber system serves largely as a support for the alveolar liquid lining layer and its associated surface tension, such that the stiffness of the septal system is affected by surface tension (Wilson and Bachofen, 1982). Lung inflation reduces interfacial surfactant which increases surface tension thus increasing the stiffness of the septa relative to that of the axial fibers. It is due to the increase in surface tension, thus in septal stiffness, that inflation shifts the duct/alveolus border outward, enlarging ducts and diminishing alveoli.

Under unphysiological conditions, surface tension effects can be more pronounced. In saline-filled lungs, Wilson and Bachofen showed that in the absence of surface tension, septa were both undulating and, due to low stiffness, expanded as the relatively stiff axial fiber system shifted the duct/alveolus border inward and reduced duct caliber. Thus, alveolar surface area has been shown by design-based stereology to be greater in saline-filled lungs lacking surface tension than in air-filled lungs with surface tension acting along the septa (Gil et al., 1979). Following removal of surfactant by lavage the high surface tension, by increasing septal stiffness relative to axial fiber stiffness, caused the duct/alveolus border to move outward such that ducts were expanded and alveoli, with essentially maximally retracted septa, very small. Accordingly, high surface tension has been shown by design-based stereology to reduce alveolar surface area (Gil et al., 1979; Bachofen and Schürch, 2001; Lutz et al., 2015; Lopez-Rodriguez et al., 2016; Knudsen et al., 2018). In lung diseases such as fibrosis and the acute respiratory distress syndrome, surface tension is elevated (Günther et al., 1999; Lutz et al., 2015; Lopez-Rodriguez et al., 2016; Nguyen and Perlman, 2020; Smith et al., 2020). Sufficiently high surface tension can dominate the axial fiber system and cause alveolar collapse and microatelectases as well as pulmonary edema (Nieman et al., 1981; Knudsen et al., 2018; Smith et al., 2020).

### 2.1 Acinar micromechanics and the glycocalyx

Recent studies draw the attention to a further structure found in the alveolar hypophase that may also influence acinar micromechanics, namely the alveolar epithelial glycocalyx observed also in co-localization with freshly secreted lamellar body-like structures and tubular myelin (Ochs et al., 2020). The epithelial glycocalyx consists of glycoproteins, proteoglycans and hyaluronan. While the proteoglycans and glycoproteins have a transmembrane domain, hyaluronan is anchored at the apical plasma membrane of the alveolar epithelial cells *via* the CD44 receptor. Targeted destruction of the alveolar epithelial glycocalyx by intratracheal heparinase in an animal model provided evidence that the interaction between surfactant and components of the glycocalyx are of relevance for the proper function of the pulmonary surfactant. The loss of the integrity of the glycocalyx led to a reduction in the surface tension lowering capacity of pulmonary surfactant as assessed *in vitro*. While simultaneous adsorption of surfactant and potential surface-tension-raising contaminants can cause artifacts during *in vitro* testing (Holm et al., 1988; Holm et al., 1999; Nguyen and Perlman, 2020; Perlman, 2020), elevated surface tension in this study was supported by a finding of decreased pulmonary compliance and the observation of instability of distal airspaces at the light microscopic level (Rizzo et al., 2022). Thus, glycocalyx degradation products should be added to the list of substances (Nguyen and Perlman, 2020) purported to raise surface tension in lung injury. Clinical investigations accentuate the relevance of glycocalyx degradation. In samples collected in filters integrated in the expiratory limb of the respirator from acute respiratory distress syndrome (ARDS) patients, Rizzo and colleagues detected increased concentrations of cleavage products of the alveolar epithelial glycocalyx such as heparan or chondroitin sulfate, particularly in patients with ARDS caused by direct lung injury. Aside from a role with regard to proper surfactant function and thus acinar micromechanics, the glycocalyx has also been suggested to be involved in regulation of the volume and viscous properties of the hypophase (Ochs et al., 2020), and mediation of inflammatory processes and regeneration (Jiang et al., 2005; Liang et al., 2016).

### 2.2 Effects of aging on the acinar microarchitecture

Alterations in the fiber network and surfactant system might be responsible for aging effects on the acinar microarchitecture and mechanics. Using hyperpolarized ^3^He gas and magnetic resonance imaging (MRI) quantitative morphological data of the pulmonary acini can be calculated in living individuals based on the model of the pulmonary acinus published by Haefeli-Bleuer and Weibel (Haefeli-Bleuer and Weibel, 1988; Osmanagic et al., 2010). In healthy non-smoking humans the diameters of the alveolar duct lumen have been shown to increase while the lengths of the interalveolar septa decrease, resulting in shallowing of the alveoli with age (Quirk et al., 2016). These finding are also referred to as ductectasia. Similar observations have been reported in aging mouse and rat lungs (Mizuuchi et al., 1994; Schulte et al., 2019). Schulte et al. described a marked increase in alveolar duct volume in 18 months compared to 12 months old mice while Ruwisch et al. (2020) observed considerable ductektasia already in much younger mice suffering from surfactant protein C (SP-C) deficiency. Quantitative data showed age dependent reorganization of the collagen and elastin fibers within the interalveolar septa such as an increase it the width of fibers combined with enlarged alveolar entrance rings, and an altered distribution within the interalveolar septa (Sobin et al., 1988). Based on computational modelling these alterations in the architecture of the fiber system can explain ductectasia, and the resulting alterations in the geometric configuration can explain the reduction in the pulmonary elastic recoil pressure due to reduced elastance of alveolar entrance rings even in the presence of stiffer septa (Subramaniam et al., 2017). The mechanisms resulting in a reorganization of the fiber system are not clear but it has been suggested to reflect adaptation. In old mouse lungs senescence markers are above all upregulated in AE2 cells and lipofibroblasts (Angelidis et al., 2019; Schiller et al., 2019). These findings are linked with data based on captive bubble surfactometer measurements providing evidence that alveolar surfactant from aged mice has an impaired ability to reduce surface tension (Yazicioglu et al., 2020). As outlined above, higher surface tension results in increased stiffness of alveolar septa, which causes septa to retract and increases septal pleating. The increased septal retraction, which reduces septal tensile load, might induce adaptation of the fiber network, and finally result in ducectasia. The observation that in SP-C knockout mice ductactasia occurs much earlier in life and before AE2 cell senescence supports this notion (Ruwisch et al., 2020).

## 3 Respiration related deformation of the lung parenchyma: Visualization and quantification

The act of breathing requires that physical forces are repetitively transmitted to the lung structures (Mead et al., 1970). These forces result in deformations, which are quantified in one, two or three-dimensions, e.g., in length, area or volume by referencing to a lower deformation state. This relative deformation is similar but not identical to strain, since the latter is referenced to an unstressed condition, which corresponds to the lung volume at zero transpulmonary pressure. The three-dimensional relative volumetric deformation of the lung is accordingly the ratio of end-inspiratory to end-expiratory lung volume (Vlahakis and Hubmayr, 2005). The stresses acting on the respiratory system result from a pressure differential between the alveolar opening and the pleural surface also referred to as the transpulmonary pressure (Mead, 1961; Mead et al., 1970; Loring et al., 2016). Forces acting on lung parenchyma are a consequence of the elastic recoil pressure. The elastic recoil pressure is caused by the surface tension at the air liquid-interface and the properties of the pulmonary fiber system consisting of elastic fibers and collagen fibrils. It has been estimated that at low lung volumes two-third of the elastic recoil pressure is due to surface tension at the air-liquid interface (Stamenović, 1990). The fiber system transmits the distending inflation pressure during inspiration to the interalveolar septa. The elastic fibers have a linear stress-strain relationship over a wide range of linear deformation so that even after doubling their baseline length the elasticity remains consistent. Elastic fibers therefore create tissue tension at low and high lung volumes. On the other hand, the collagen fibrils within the interalveolar septa have a ‘curly’ configuration at low lung volumes. With pulmonary inflation these fibrils are progressively straightened and become stress bearing, bringing their rigid mechanical properties into play to limit distension. Therefore, collagen fibrils become stress bearing at larger lung volumes, e.g., above 80% of TLC (Suki et al., 2005; Bou Jawde et al., 2020). A similar micromechanical behavior has been proposed for the BM located in the blood-gas barrier (Maina and West, 2005). Among others, the BM is composed of type IV collagen, proteoglycans, laminin, integrins and other anchoring fibers involved in producing cell-matrix junctions, e.g., with the alveolar epithelial cells. The type IV collagen located within the lamina densa appears to be of high relevance to the micromechanical properties of the BM. Here, it has been suggested that dimers of type IV collagen molecules form a network of rhombic meshes with an edge length of approximately 800 nm (Timpl et al., 1981). This network of type IV collagen dimers allows some degree of deformation as indicated by changes of the surface area of the BM measured at different lung volumes which shows two-dimensional strain (Tschumperlin and Margulies, 1999). At the same time, the BM has given proof of its resilience against stress failure in several studies. While injurious mechanical ventilation destroys the alveolar epithelial cells in rodent lungs (the AE1 much more than the AE2 cells!), the ultrastructure of the underlying BM remains preserved (Dreyfuss and Saumon, 1998; Albert et al., 2020). Notably, at low lung volumes, the blood-gas barrier including the epithelial BM creates pleats and these pleats are recruited with inspiration (see below) so that the BM together with the attached epithelial cells become planar. Hence, it seems reasonable that BM becomes stress bearing at larger lung volumes and contributes to lung mechanical properties measured at the organ scale similarly to the collagen fibrils within the interalveolar septa discussed above (Maina and West, 2005). An intact surfactant system and the elasticity of the scaffold of the interalveolar septa protect the cellular components of the blood-gas barrier, above all the AE1 cells, from injurious strain during tidal ventilation. These counterbalanced forces keep the cells from bearing substantial stress within the physiological range of breathing (Wilson and Bachofen, 1982). The observation that the mechanical properties of the lung hardly change during the process of de-cellularization supports this notion (Nonaka et al., 2014).

It is well known that there is some heterogeneity in ventilation, and therefore strain, within the human lung at macroscopic scale, a feature that has recently been observed in mice and rats as well (Arora et al., 2021). At the microscopic level such as within an acinus, however, heterogeneity, appears to be avoided. Because neighboring alveoli are divided by shared septa and the network of stress-transferring structures running throughout the interalveolar septa interconnects numerous alveoli, the mechanical properties of the alveoli are interdependent. Impaired mechanics, e.g. due to alveolar flooding of one alveolus, have an impact on the mechanics of adjacent alveoli and deformation/stretching of those bounding septa creates a stress concentration (Perlman et al., 2011). In a healthy lung, however, the pulmonary surfactant system not only reduces but also harmonizes surface tension in the alveoli, so that alveoli of different sizes can co-exist and stress concentration is avoided (Schürch, 1982; Schirrmann et al., 2010). Low volume mechanical ventilation or spontaneous breathing in presence of stress concentrations, such as microatelectases or flooded alveoli, results in injury of the blood-gas barrier and degradation of lung mechanics, despite no increase in strain at the organ level (Wu et al., 2014; Albert et al., 2020; Krischer et al., 2021; Bachmann et al., 2022). High tidal volume ventilation with low positive end-expiratory pressure produces progressive ventilation-induced lung injury with severe damage of the blood-gas barrier in mice (Hamlington et al., 2018). Analysis of the progression of this ventilation-induced injury reveals that cellular injury initially forms in quasi-random locations. Continued ventilation causes those initial points of injury to spread locally so that the number of damaged cells in an “injury cluster” grows with continued ventilation, with the initially injured lung regions acting as seeding point. As such, injury occurs predominantly at the interface between injured and healthy lung regions and this can be explained by stress concentration and alveolar interdependence (Cereda et al., 2017; Mattson et al., 2022).

In the healthy lung, the biomechanical properties of the interalveolar septa are relatively homogenous so that ventilation (strain) and parenchymal stress are also quite homogenous, preventing stress concentrations and subsequent stress failure of the delicate structures in the blood-gas barrier (Mead et al., 1970; Makiyama et al., 2014; Albert et al., 2019; Perlman, 2020). Given that lung parenchyma of an average human must withstand approximately 10^9^ low- and 10^7^ higher-volume (e.g. exercise, deep sighs) breathing cycles it becomes obvious that the avoidance of local stress concentration is of high importance (Fredberg and Kamm, 2006).

Generally, stresses in the acinus result in deformation of the acinar airways and interalveolar septa. The mechanisms of tissue deformation that accompany ductal and alveolar airspace volume changes are quite different due to the structural arrangement of the surrounding tissues. Volumetric strain within the alveolar duct is linked with deformation of the mesh-like network of alveolar entrance rings forming its boundary, while strain of alveoli requires adaptation of the interalveolar septa. Within the interalveolar septa, strains are imposed on both the lumen of alveolar capillary network and the blood-gas barrier. The cellular components of the blood-gas barrier are particularly vulnerable to injury caused by excessive deformation. A two-dimensional cyclic strain of 25% applied to alveolar epithelial cells has been shown to induce cellular injury and apoptosis in *in vitro* model systems, emphasizing the vulnerability of these cells and thus the need to protect them (Tschumperlin et al., 2000; Dolinay et al., 2017).

Recent advances in clinical imaging now allow visualization of some aspects of the spatial heterogeneity discussed above. For instance, dual energy computed tomography has been applied to investigate regional ventilation (volumetric strain) within the lung parenchyma in spontaneously breathing patients suffering from idiopathic pulmonary fibrosis (IPF), a progressive, scarring lung disease with limited prognosis. Scharm and co-workers used end-inspiratory and end-expiratory scans to quantify regional volume changes (Scharm et al., 2021). IPF is characterized by an increased heterogeneity of regional ventilation compared to healthy subjects. The ventilation heterogeneity is highly correlated with future decline in lung function, and the regions with increased strain are at the highest risk for fibrotic remodeling (Scharm et al., 2022). In other words, abnormalities in regional ventilation precede fibrotic remodeling so that it is tempting to hypothesize that excessive strain of fine lung parenchyma (i.e. abnormal acinar micromechanics) is a trigger for the formation of scars and degradation of lung function (Knudsen et al., 2017; Albert et al., 2019), a concept which has recently been supported in mice (Beike et al., 2019; Wu et al., 2020). Nevertheless, the exact abnormalities at the micromechanical level responsible for these clinical imaging-based observations remain unclear.

To investigate all aspects of acinar micromechanics during spontaneous breathing or invasive mechanical ventilation, real-time visualization of the affected structures in three dimensions is desirable. Intravital microscopy has been applied to study the dynamics of subpleural alveoli, providing valuable insights into to changes in alveolar dimensions, capillary network perfusion, and oxygenation during mechanical ventilation (Matuszak et al., 2020; Masterson et al., 2021). Under physiological conditions, changes in alveolar volume occurs in synchrony with the respirator (Tabuchi et al., 2016) and the alveolar volume changes are comparably small so that alveolar volumetric strains are low with physiological tidal volumes (Schiller et al., 2003). Linear strain of alveoli in the volume range of quiet spontaneous breathing has also been estimated using lungs fixed at different lung volumes and quantitative microscopy. These strains range from 4% (Mercer et al., 1987; Tschumperlin and Margulies, 1999; Roan and Waters, 2011) to 10% (Gil et al., 1979). Imaging the same alveoli at different airway pressures with confocal microscopy in an *ex vivo* model-system resulted in similar findings: increasing pressure from 5 to 10 or 15 cmH_2_O was linked with an increase in alveolar perimeter of less than 5 or 10%, respectively. Moreover, alveoli expanded non-uniformly with septal surface covered by AE1 cells deforming more than that covered by AE2 cells (Perlman and Bhattacharya, 2007), an observation supported by design-based stereological investigations at the electron microscopic level during deflation from 10 to 2 cmH_2_O (Ruhl et al., 2019).

However, the penetration depth of intravital microscopy is limited to subpleural alveoli. The relevance of this limitation is highlighted in a recent study that used confocal microscopic imaging of cleared lungs to show that the subpleural parenchymal architecture differs from other regions of the lung in diverse species (including humans) (Mitzner et al., 2020). The alveolar ducts run perpendicular to visceral pleural surface so that the region immediately beneath the pleura contains a single layer of alveoli with boundaries and numbers of neighboring alveoli that significantly differ from regions deeper in the lung. Hence, the micromechanics of subpleural alveoli are likely to differ from other regions.

Micro-computed tomography or synchrotron-based tomography have sufficient resolution to resolve interalveolar septa in more central regions of the lungs. Accordingly, Sera and co-workers imaged mouse lungs repetitively at different degrees of quasi-static inflation with synchrotron refraction enhanced computed tomography to describe a complex, accordion-like expansion of the acini. Moreover, at lower lung volumes up to an airway opening pressure of 8 cmH_2_O, strain appeared to be larger in the alveolar ducts than in the alveoli. At higher lung volumes both of these compartments are subject to approximately equal deformations (Sera et al., 2013). Similar results have been reported based on three-dimensional reconstructions of alveoli using serial sections from lungs fixed at different lung volumes during expiration (Mercer et al., 1987). Those studies determined acinar micromechanical behavior under quasi-static conditions. However, due to the viscoelastic properties of the parenchyma it is likely that under physiological, dynamic breathing the micromechanical properties are different.

Advances in imaging methodology including tracking X-ray tomography (Chang et al., 2015) and synchrotron-based, phase-contrast micro computed tomography (Bayat et al., 2022) now allow study of dynamic processes within the acinus. Chang and co-workers tracked profiles of alveoli in different lung regions of mice during spontaneous breathing by X-ray tomography. Inflation was heterogeneous, resulting in a dynamic linear septal strain of 5.7% in apical and 8.7% in basal lung region. Overall, approximately 1/3rd of tidal volume was delivered to the alveolar compartment and 2/3rd to alveolar ducts (Chang et al., 2015). These estimations align with the findings under quasi-static conditions mentioned above (Sera et al., 2013). Cercos-Pita and colleagues used synchrotron-based dual energy micro computed tomography to image acinar airspaces of mechanically ventilated rats with a high temporal resolution of 78 three-dimensional datasets per breath. At a tidal volume of 8 ml/kg (PEEP = 6 cmH_2_O, peak inspiratory pressure = 12 cmH_2_O) the relationship between acinar volume (V_A_) and surface area (S_A_) given by the formula S_A_ = k V_A_^n^ suggests distension of alveoli as the predominant mechanism of the acini to accommodate to volume changes (Cercos-Pita et al., 2022). This discrepancy might be explained by differences in the protocols, e.g. investigating spontaneously breathing animals or mechanically ventilated animals with higher PEEP. Although synchrotron-based micro computed tomography is a very powerful imaging approach to investigate acinar micromechanics, access to the required equipment is limited.

Imaging techniques with a resolution at the light microscopic level are appropriate to investigate alterations in alveolar and ductal airspaces. However, the different compartments of the interalveolar septa are generally beyond the resolution of light microscopy or x-ray-based imaging modalities. During the respiratory cycle, those components of the interalveolar septa have to adapt to volume changes within the airspaces. Based on investigations including morphometry using light and transmission electron microscopy of lungs fixed at different lung volumes in the expiratory limb of a pressure-volume loop, investigators suggested several adaptive mechanisms of alveoli and subsequently of the interalveolar septa. These include 1) recruitment and derecruitment of complete alveoli, 2) recruitment and derecruitment of parts of alveoli by folding and unfolding of pleats of the interalveolar septa, 3) balloon-like change in alveolar size by stretching and de-stretching of the septal tissue (including the blood-gas barrier) and 4) alteration in the alveolar shape without stretching/de-stretching or recruitment/derecruitment of folds (Gil et al., 1979; Wilson and Bachofen, 1982; Bachofen et al., 1987; Knudsen and Ochs, 2018).

Aside from alveolar recruitment and derecruitment, which has been observed directly by investigators using *in vivo*/intravital microscopy, particularly in acutely injured lungs (Carney et al., 1999; Schiller et al., 2001; Halter et al., 2007; Pavone et al., 2007), it is difficult to visualize the other mechanisms using real-time imaging. Those mechanisms predominantly involving the blood-gas barrier, such as folding/unfolding or de-stretching/stretching, require electron microscopic resolution to identify the AE1 cells and the underlying BM (Bachofen et al., 1987; Tschumperlin and Margulies, 1999; Ruhl et al., 2019). In order to quantify the contribution of the different mechanisms to the adaptation of alveoli and their walls to changes in lung volumes, quantitative morphology based on design-based stereology and electron microscopy is the method of choice (Hsia et al., 2010; Ochs and Schipke, 2021). The stereological parameters useful for quantification of alveolar recruitment and derecruitment, recruitment and derecruitment of pleats, and stretching of the BM are summarized in the Table and will be introduced in more detail in the following sections.

### 3.1 The concept of design-based stereology

Design-based or unbiased stereology is founded on stochastic geometry. It derives three-dimensional data from two-dimensional sections and can be applied to any imaging modality (Knudsen et al., 2021). This section provides a brief introduction into the concepts of design-based stereology. Afterwards, we will summarize the available literature in which design-based stereology has been used to investigate acinar micromechanics with a focus on the septa.

In order to analyze the fine three-dimensional structure of biological samples, nearly two-dimensional sections are classically visualized using microscopy. This approach holds some problems because the structures are three-dimensional and one dimension is lost when the investigation is performed in two-dimensions. A second problem is the fact that the nearly two-dimensional sections represent only a small fraction of the entire organ. Hence, the observations might not be representative. A third problem is called the “reference trap.” Consider the case where the lungs of two study groups are to be compared with each other. One study group suffers from high surface tension which results in an instability of alveolar airspaces so that microatelectases occur, there is less air inside the lungs, and lung volumes are 1/3 smaller. Light microscopic quantification reveals that 35% and 25% of lung volumes are comprised of interalveolar septa in the high surface tension and control groups, respectively. This might lead to the erroneous conclusion that there is more septal tissue in the group suffering from high surface tension, which is not the case since the lung volumes (the *reference space*) are different. Multiplication of the volume fraction of interalveolar septa (35% and 25%, respectively) with the associated lung volumes yields the absolute volumes of interalveolar septa per lung and reveals whether or not a difference exists. To avoid this reference trap, the reference volumes must be determined and quantitative structural data need to be related to the reference volume (Tschanz et al., 2014).

**FIGURE 4:**
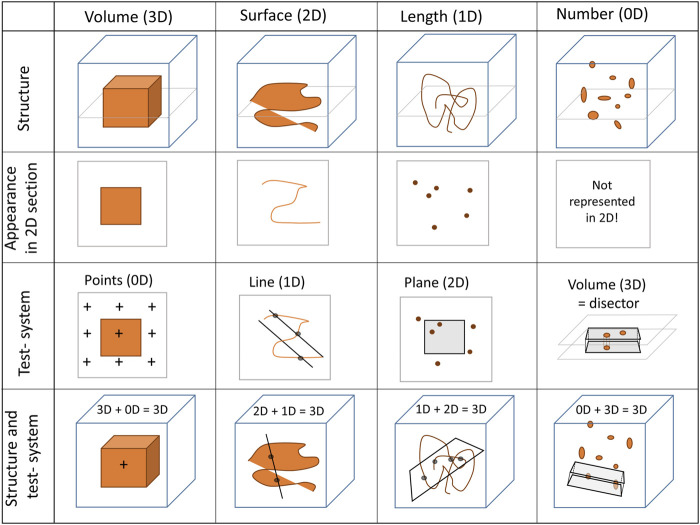
Stereological test-systems: On two-dimensional (2D) sections, three-dimensional structures (3D) lose one dimension. Accordingly, a volume appears as an area (2D), a surface area as a line (1D) and a length as a point (0D). The numerical quantity of a structure is a dimensionless (0D) parameter in three-dimensional space. Since a negative dimension is not possible this parameter is not represented on two-dimensional images. Hence, quantity of any structure cannot be determined from single sections based on the principles of stochastic geometry (or any other method)—an unbiased test-volume (3D) generated by a disector is required.

Design-based stereology, which is predicated on mathematical principles, is an efficient solution for the problems mentioned above. It does not make any assumptions considering the orientation, size, spatial distribution or shape of the structures under study. Hans Elias, who pioneered the development of stereology in biomedical research, expressed the following definition for stereology: “extrapolation from two to three-dimensional space, or three-dimensional interpretation of two-dimensional images, by methods of geometric probability” (Elias, 1971). Although two-dimensional images are used, stereology provides three-dimensional, representative and unbiased data of structures of interest. To get three-dimensional information from two-dimensional images, geometric test-systems are randomly superimposed on randomly sampled images and interact with the structures of interest (e.g., the surface area of the BM) in a stochastic way. The dimension of the structure of interest and the dimension of the test-system always sum up to 3. For example, the surface area of the epithelial BM (a two-dimensional parameter) is assessed with a test system comprised of one-dimensional lines. Here, the test-lines are projected onto randomly selected images and interact with the surface of the BM by creating intersections. On the two-dimensional sections, the surface area of any structure has lost one dimension so that a surface appears as a line. The number of counted intersections between the test-lines and surface boundary is a stochastic value and may have slightly different values each time the process is repeated due to the randomization of images and line placements. Nevertheless, the expected number of line-surface intersections is directly proportional to the length of the test-line (which is known) and the density of BM surface area per volume of the reference space (e.g., the volume of the lung). The same rules apply to determination of volumes (e.g., the volume of interalveolar septa), lengths (e.g., the length of blood vessels), and numbers (e.g., the number of AT2 cells) which require test-points, test-areas and test-volumes for counting, respectively (Figure 4). Practical “guidelines” how to use design-based stereology in lung research have been reviewed in several publications, e.g., (Mühlfeld et al., 2013; Tschanz et al., 2014; Mühlfeld et al., 2015).

To guarantee that stereological data are unbiased the tissue processing and sampling steps are critical. Every part of the organ must have the same chance of being investigated. For example, quantification of the number of alveoli per lung requires that each alveolus, independent of its features such as size or orientation, must have the same probability of being counted. An unbiased stereological design achieves this requirement by randomization at each step of the study, starting with study subject and ending with the projections of the test-systems on the randomized fields of view for counting. Several methods for randomization of tissue samples have been published (Ochs and Schipke, 2021). A further source of bias is changes in tissue dimensions during the processing steps for microscopy. In particular, paraffin embedding results in large, unpredictable, and heterogeneous shrinkage (up to 50%–60% by volume). Osmification and embedding in plastic (e.g. glycol methacrylate or epoxy resin) is preferred for quantification of lung structure because this approach prevents tissue shrinkage (Schneider and Ochs, 2014). When studying acinar micromechanics by comparing data from lungs fixed at different degrees of lung inflation heterogenous shrinkage in critical because the *in vivo* dimensions of the structures must be preserved. A limitation of quantitative morphology including design-based stereology is that there is no optimal fixation technique, e.g., a gold standard available to preserve the structure in a way that reflects all aspects of the *in vivo* architecture. If, for example, perfusion fixation is used to preserve the airspace morphometry then the dimensions of the vascular structures are dependent on the perfusion pressure.

Nevertheless, to obtain valid data from stereological investigation the fixation technique and the fixation solution can be optimized for the structures of interest. As pointed out earlier, surface tension and the elastic fiber system are important contributors to micromechanical behavior of the acini. Therefore, when studying the dimensions of the airspace and interalveolar septa the lungs need to be preserved in a way that reliably fixes the elastic fibers and preserves the shaping effect of surface tension on the acinar microarchitecture. Moreover, fixation needs to be performed at an airway opening pressure which is of relevance for the given research question, e.g., within the range of transpulmonary pressures used for mechanical ventilation. This goal can be achieved by vascular perfusion, which preserves the hypophase including the intra-alveolar surfactant. In their guidelines paper, Hsia and co-workers suggested fixing the lungs by vascular perfusion with glutaraldehyde, osmium tetroxide (OsO_4_), and ethanol to maintain the surface liquid layer and lung volume (Bachofen et al., 1982; Hsia et al., 2010). The glutaraldehyde fixes tissue by crosslinking proteins whereby maintaining the ultrastructure for electron microscopic investigation. However, it does not entirely fix the elastic fibers. Thus the remaining elastic recoil might result in volume loss during lung preparation (Oldmixon et al., 1985). The additional perfusion of the pulmonary vasculature with OsO_4_ and ethanol fixes the elastic fibers more reliably and thereby stabilizes the lung volume. In most cases the perfusion with OsO_4_ is omitted due to the high toxicity and rather small effects of the remaining elastic recoil on lung volume after glutaraldehyde fixation, which has been estimated ≈2% in linear dimensions in rat lungs (Tschumperlin and Margulies, 1999; Knudsen et al., 2018).

Design-based stereology has been employed in several studies to analyze acinar micromechanics using lungs fixed under quasi-static conditions representing different degrees of inflation up to TLC or deflation coming down from TLC (Gil et al., 1979; Bachofen et al., 1987; Knudsen et al., 2018; Smith et al., 2020). The total lung capacity corresponds to a lung volume at transpulmonary pressure of 25–30 cmH_2_O. The range of physiological, quiet breathing corresponds to 40%–80% of TLC and is located in the linear portion of the pulmonary pressure-volume curve (Suki et al., 2011). Here, the transpulmonary pressures needed to reach a certain lung volume differ between the inspiratory and expiratory limb. On expiration, the range of transpulmonary pressures to reach 40%–80% TLC is roughly between 3–4 and 8–10 cmH_2_O in rats, rabbits and mice (Bachofen et al., 1987; Tschumperlin and Margulies, 1999; Lai and Chou, 2000). Lungs may be fixed either *in vivo* or *ex vivo*, and in both cases careful control of the lung volume is critical. Under *ex vivo* conditions a drop of the lung volume below residual volume must be avoided since this introduces unphysiological conditions (e.g., complete or partial lung collapse) that reflect in lung structure. *Ex vivo*, the airway opening pressure equals the transpulmonary pressure. *In vivo*, however, the calculation of the transpulmonary pressure requires the knowledge of the pleural pressure or a thoracotomy. How the airway opening pressure translates to the transpulmonary pressure in anesthetized mice has been described by Lai and Chou (Lai and Chou, 2000).

### 3.2 The acinar airspaces: Alveolar and alveolar duct compartment

**FIGURE 5:**
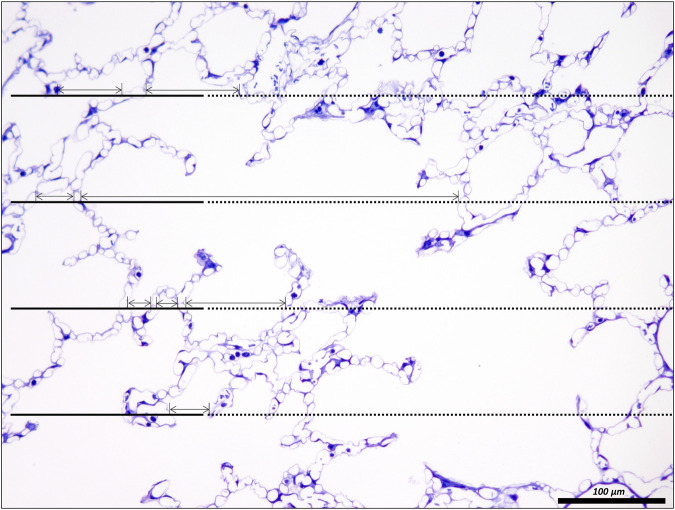
Unbiased test-system for measurements of chord length of acinar air spaces. A healthy rat lung was fixed *in vivo* by vascular perfusion *via* the vena cava caudalis at an airway opening pressure of 5 cmH_2_O on expiration after two recruitment maneuvers (3 s pause at 30 cmH_2_O) (Knudsen et al., 2018). The parameter “chord length” is also known as the linear intercept length. It is based on simple, linear measurements of the dimension of the acinar airspaces from one border to the next. The left side of the randomly sampled image contains four straight line-segments, extended to the right by a dashed line, the so-called guard line. The line segments on the left serve to sample the starting point of the measurements. Each time the line segment intersects an interalveolar septum a measurement is performed from the intersection to the next surface of an interalveolar septum. The direction of the measurement follows the run of the test line and if needed also the dashed guard line. In order to locate the points of measurements exactly the top border of the line segment is used. The arrows label the measurements in this example. Some measurements are performed within an alveolus, others, however, travers *via* the alveolar opening through the alveolar duct airspace to the other side so that these measurements encompass both alveolar and alveolar duct airspaces.

**FIGURE 6:**
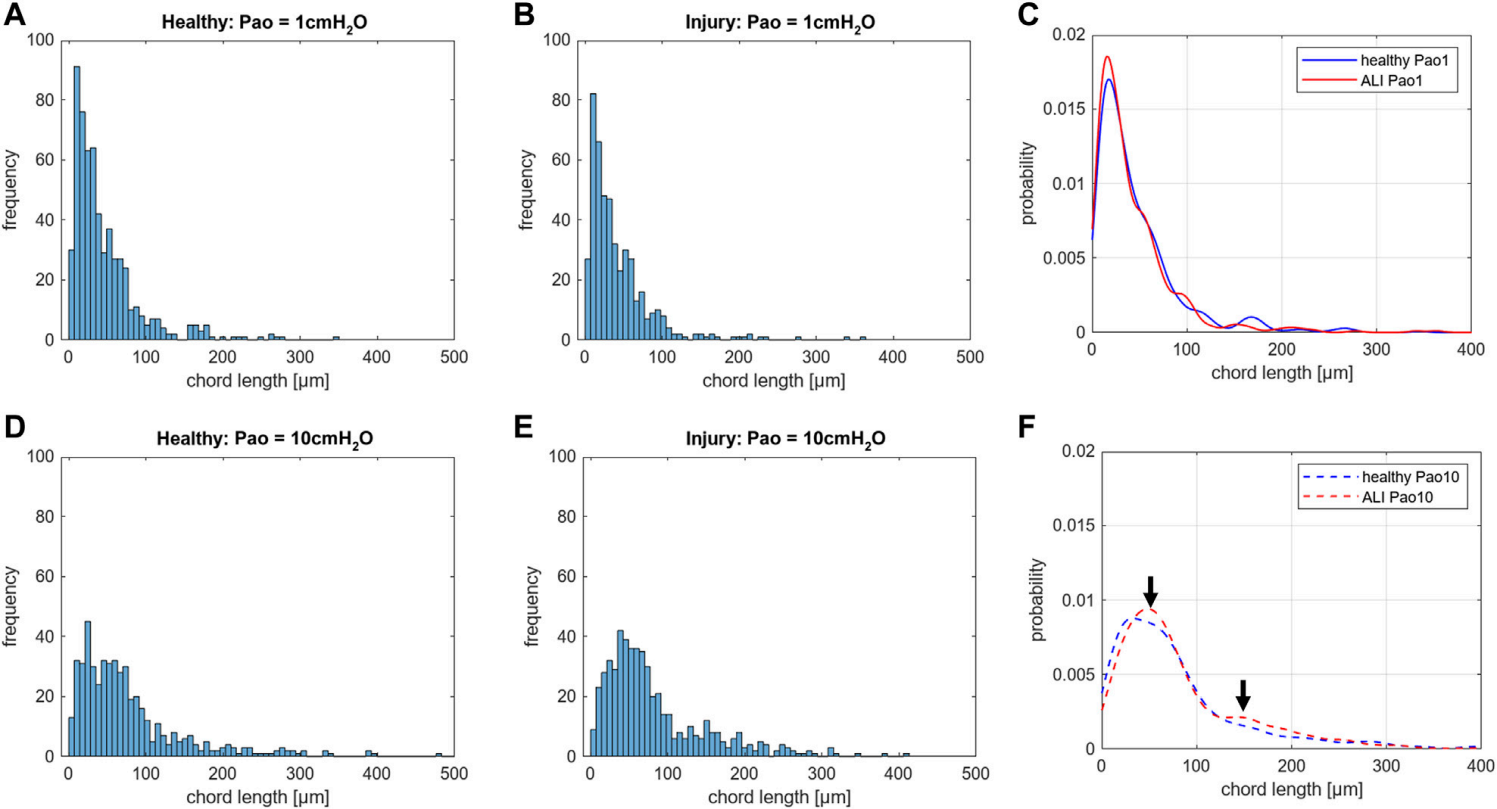
Distribution of chord lengths of acinar airspaces: Healthy and injured (ALI) rat lungs were fixed *in vivo* by vascular perfusion *via* the vena cava caudalis at an airway opening pressure (Pao) of either 1 cmH_2_O on expiration or 10 cmH_2_O on expiration after two recruitment maneuvers (3 s pause at 30 cmH_2_O) (Knudsen et al., 2018). The distributions of chord lengths of acinar airspaces are illustrated as histograms and probability based on the Kernel probability distribution function. In both healthy and injured lungs fixation at higher Pao on expiration results in a right shift of the measurements. At Pao = 1 cmH_2_O, hardly any differences can be identified in the histograms between healthy **(A)** and injured lungs **(B)**. Accordingly, the Kernel probability distribution function shows hardly any differences **(C)**. At Pao = 10 cmH_2_O, the histograms suggest a right shift of the peak in the injured lung **(E)** compared to the healthy lung **(D)**. The Kernel probability distribution function supports this right shift and indicates a second peak at larger chord length in the range of 150 µm **(F)**. Note, the investigation was performed at a very early time point of bleomycin-induced acute lung injury development at which lung mechanical measurements were scarcely affected. In each group, 500 measurements were performed on randomized fields of view from two lungs.

The acinar airspaces are subject to cyclic volume changes during respiration. In three-dimensional datasets from, e.g., micro computed tomography or synchrotron-based imaging, appropriate image processing protocols can segment acini and compute their surface area and air volume semi-automatically (Vasilescu et al., 2012; Haberthür et al., 2021). These data provide valuable information on deformation when determined at different levels of inflation or (preferably) dynamically during ventilation (Cercos-Pita et al., 2022). Using two-dimensional images, which are oftentimes more readily obtainable, an efficient and easy tool to investigate the dimensions of the acinar airspaces is the determination of chord lengths (= linear intercept length), which are randomized, linear measurements from one boundary of the acinar airspace to the next (Knudsen et al., 2010). Although it is an one-dimensional parameter describing a complex three-dimensional anatomical structure, it can easily be determined with the help of test-lines projected on the randomized fields of view (Figure 5). An advantage is that chord length measurements also provide information of lung function in terms of the free path of oxygen to the alveolar wall as well as airspace heterogeneity occurring with different lung volumes. The chord length of acinar airspaces correlates well with the apparent diffusion index of hyperpolarized helium determined with magnetic resonance tomography (MRI) (Woods et al., 2006), an *in vivo* measurement that also gives information on regional mechanics and microarchitecture (Choy et al., 2010). Chord length measurements can be performed with non-destructive imaging including micro computed tomography as well as MRI (Chan et al., 2021). In healthy lungs and early bleomycin-induced acute lung injury fixed *in vivo* at stable airway pressures of 10 or 1 cmH_2_O on expiration (Lutz et al., 2015; Knudsen et al., 2018), the distribution plots of chord lengths are comparable at low lung volumes (Figure 6). With higher lung volume the heterogeneity of chord lengths increases, a pattern which is more pronounced in lung injury. The most frequently observed intercept length (peak of the histogram) is shifted to the right (larger lengths) in the injured compared to the healthy lungs. Also, a second peak is unmasked in the injured but not in the healthy lung in the range of 150 µm. Thus, the dimensions of the acinar airspaces become more heterogeneous in injured lungs at higher pressures although at that very early timepoint after bleomycin the injury is subtle. These observations in the distribution of chord lengths might indicate abnormalities in acinar micromechanics resulting in increased ventilatory heterogeneity, a mechanism which has been discussed to contribute to ventilation-induced lung injury (VILI) *via* mechanical stress (Albert et al., 2019; Nieman et al., 2020).

**FIGURE 7:**
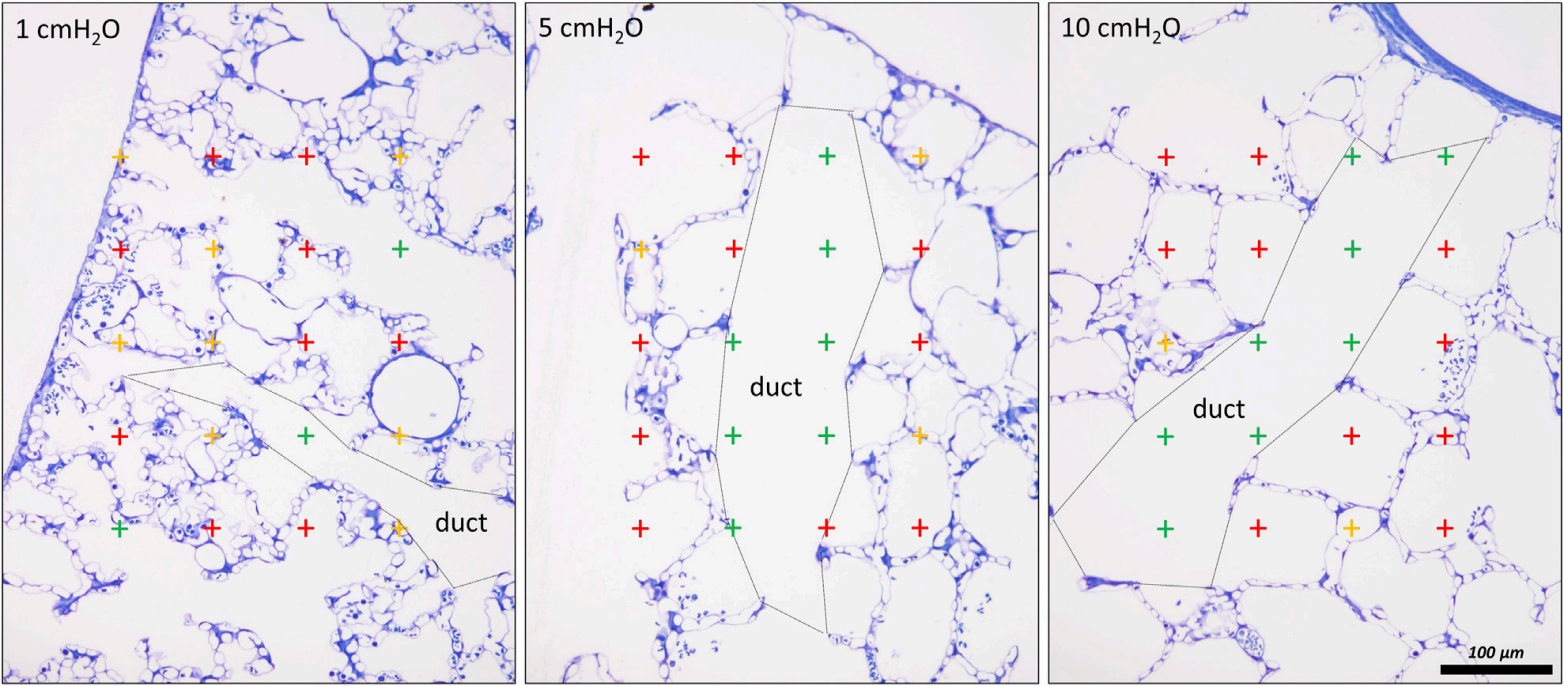
Differentiation of acinar airspaces by point counting: Healthy rat lungs were fixed *in vivo* by vascular perfusion *via* the vena cava caudalis at airway opening pressures of either 1, 5, and 10 cmH_2_O on expiration after two recruitment maneuvers (3 s pause at 30 cmH_2_O) (Knudsen et al., 2018). In order to determine the volume fractions of tissue, alveolar or alveolar duct airspaces within the lung, test points were superimposed on randomized fields of view. Lungs were treated by immersion in 4% OsO_4_ before dehydration and embedded in glycol methacrylate to avoid shrinkage/tissue deformation after fixation. The probability of a test point hitting the profile of a structure of interest is directly proportional to the volume fraction of this structure of interest within the reference space. The ratio of test points hitting the structure of interest and the reference space provides the volume fraction of the structure of interest. In the examples, the probability of a test point placed on the randomly sampled fields of view depends on the volume fraction of tissue, alveolar airspace or alveolar duct airspace within the lung. The multiplication of the volume fractions with the lung volume will result in the absolute volumes of tissue, alveolar airspace or alveolar duct airspace per lung. With the goal to separate alveolar duct and alveolar airspace, the entrances into the alveoli were closed by drawing a straight line between the edges of the interalveolar septa. Points hitting alveolar duct airspace (green), alveolar airspace (red) and tissue (yellow) were labelled in the examples. At low Pao, the alveolar ducts were small and the inter-alveolar septa appeared to be at rest with a curvy or in part crumpled surface. At larger Pao, the alveolar ducts widened considerably, the inter-alveolar septa straightened (and appeared to be under tension) and the alveoli became larger. The number of test points hitting alveolar as well as alveolar duct airspaces increased on the expense of the tissue. If the images were representative for the whole organ, one would assume that the volume fractions of tissue deceases with inflation pressure. In order to be representative, however, it is advised to count 100—200 hits on a structure of interest from at least 60 randomized fields of view sampled from at least four randomized sections per organ.

The chord length measurements, however, do not differentiate between alveolar and ductal airspaces. The strain is different in these two compartments and depends on the considered range of lung volumes (Mercer et al., 1987; Sera et al., 2013; Chang et al., 2015; Cercos-Pita et al., 2022). In healthy lungs, at lower lung volumes, volume changes manifest predominantly within the alveolar duct compartment. In contrast, at larger lung volumes with transpulmonary pressure above 8–10 cm investigations suggest that volume changes occur in alveolar and ductal airspaces alike, or even predominantly in the alveolar compartment. During acute lung injury due to VILI or intratracheal bleomycin several studies demonstrated that loss of volume primarily happens in the alveolar compartment while the alveolar duct compartment remains stable or even increases in size (Lutz et al., 2015; Smith et al., 2020). Thus, it makes sense to quantify these two compartments separately to provide a more nuanced description. Figure 7 shows micrographs of healthy lungs fixed *in vivo* by vascular perfusion at airway opening pressures of 1, 5 and 10 cmH_2_O on expiration (Knudsen et al., 2018). In order to separate alveolar and ductal airspaces, straight lines are drawn between the free edges of the interalveolar septa, representing the alveolar entrance rings. The volume fraction of these two compartments in the lung parenchyma is then determined by applying a stereological test system of test points to randomized micrographs and counting points falling on alveolar or alveolar duct airspace. On expiration, the ratio of the volumes fractions of alveolar airspaces (V_A_) to alveolar duct airspaces (V_D_) is roughly stable in the range of 1.5–2 at airway opening pressures above 10 cmH_2_0. This indicates that during deflation, and under quasi-static conditions, the absolute volume changes in the alveolar compartment is 1.5–2 times larger than that one found in the alveolar duct compartment keeping the V_A_/V_D_ ratio stable. As pressures drop below 5 cmH_2_O the V_A_/V_D_ ratio increases up to 2.8–4, so that volume loss is higher in alveolar duct compartment (Mercer et al., 1987; Knudsen et al., 2018).

In lung injury models, the absolute volume of the alveolar duct compartment remains stable or increases so that the V_A_/V_D_ ratio is in general smaller over a wide range of airway opening pressures (Knudsen et al., 2018; Beike et al., 2019; Smith et al., 2020; Rizzo et al., 2022). The expansion of the alveolar ducts can best be explained by the Wilson- Bachofen model: lung injury related high surface tension retracts the alveolar septa so that the duct volume increases (Bachofen et al., 1979; Wilson and Bachofen, 1982).

### 3.3 Interalveolar septa

Injury-induced alterations in the alveolar compartment often demonstrate strong correlations with abnormalities in lung mechanics. For example, the degree of alveolar collapse highly correlates with the pulmonary system elastance as shown in several studies (Lopez-Rodriguez et al., 2016; Steffen et al., 2017; Beike et al., 2019; Smith et al., 2020). In healthy lungs, the decline in mean alveolar size at low lung volumes correlates highly with an increase in pulmonary system elastance measured at corresponding pressures (Knudsen et al., 2018). The alterations in the alveolar compartment are accompanied by deformations of the interalveolar septa including the recruitment/derecruitment of septal pleats, stretching/un-stretching of the tissue, and shape changes without stretching. These biomechanical modifications of the interalveolar septa all appear to contribute to lung mechanical function in different ways.

#### 3.3.1 Folding and unfolding of septal pleats

**FIGURE 8:**
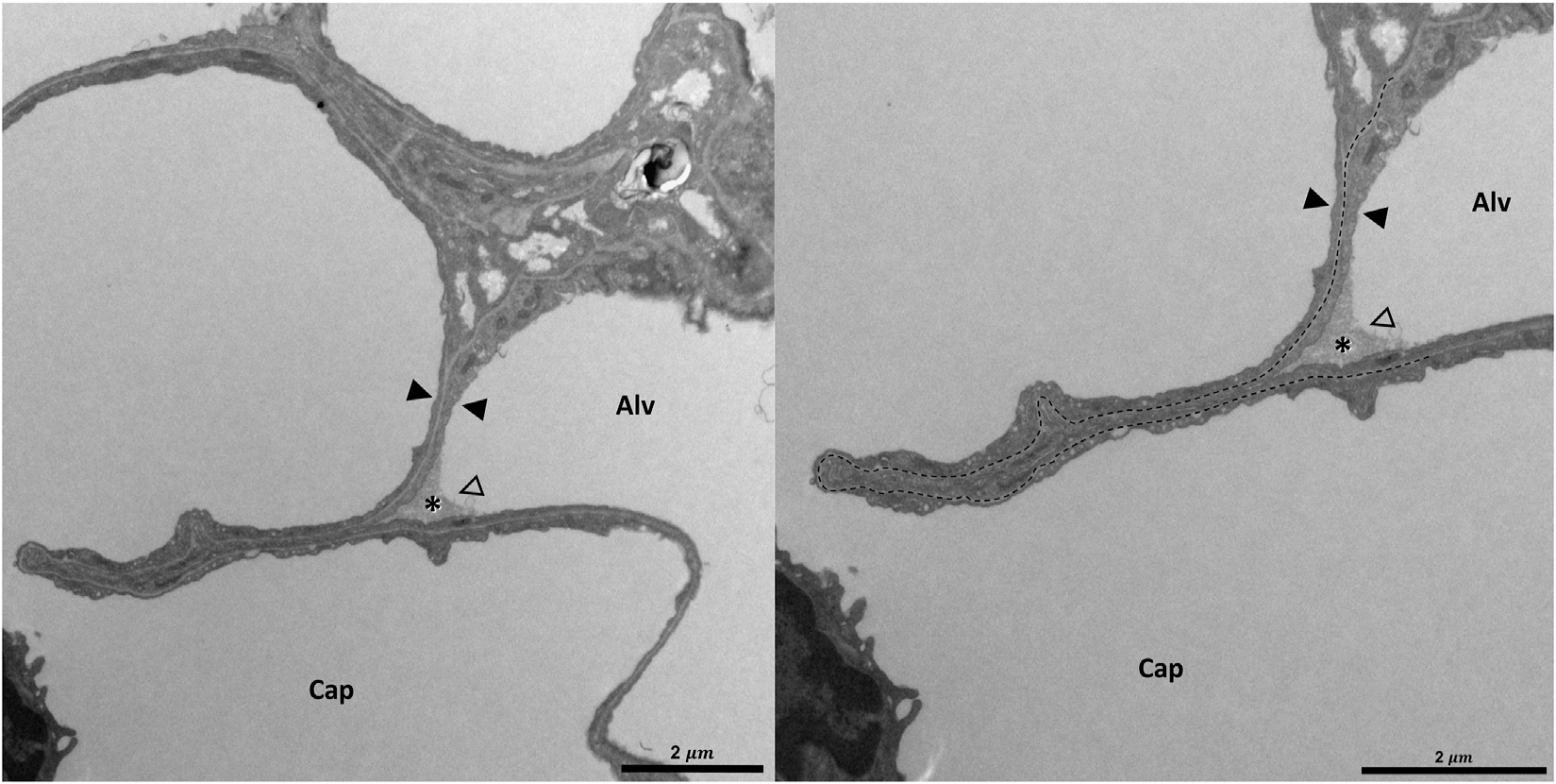
Pleating of the blood-gas barrier: A healthy rat lung was fixed *in vivo* by vascular perfusion *via* the vena cava caudalis at an airway opening pressure of 5 cmH_2_O on expiration after two recruitment maneuvers (3 s pause at 30 cmH_2_O) (Knudsen et al., 2018). The capillary network is open and nearly free of blood cells. The empty arrowhead points to the entrance of a pleat that is filled with a protein containing fluid (asterisk). Pleats are generally filled with a small quantity of proteinaceous fluid but occasionally locations of direct epithelial-epithelial contact are observed. The pleat is limited to the blood-gas barrier, which invaginates into a capillary (Cap). Two filled arrowheads locate the blood-gas barrier. The image on the right shows the run of the basement membrane (black dashed line), shared by the endothelial cell and the alveolar epithelial type 1 cell, within the pleat.

**FIGURE 9:**
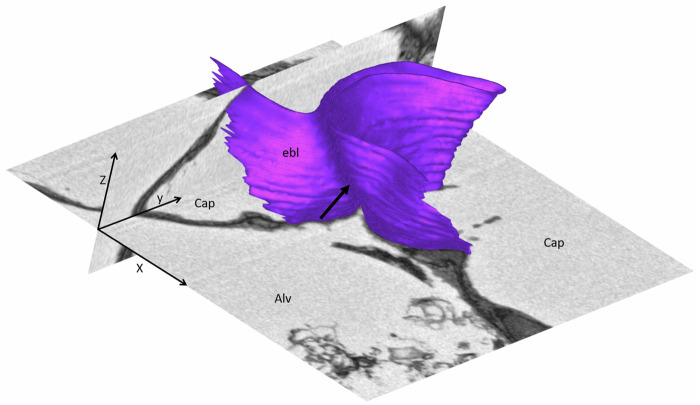
Three-dimensional model of a pleat: The mouse lung was fixed *in situ* by vascular perfusion *via* the right ventricle at an airway opening pressure of 2 cmH_2_O on expiration after two recruitment maneuvers (3 s pause at 30 cmH_2_O) (Ruhl et al., 2019) and processed for serial block-face scanning electron microscopy (SBF-SEM) (Buchacker et a. 2019). The EM image stack was used to segment the shared basement membrane of the endothelial and alveolar epithelial type I cell (ebl, magenta) within a pleat. The arrow points at the slit-like entrance to the pleat, which is created by the blood-gas barrier and invaginates sickle-shaped into the capillary (Cap). The model of the pleat is put into the context of the EM stack by two-dimensional images.

**FIGURE 10:**
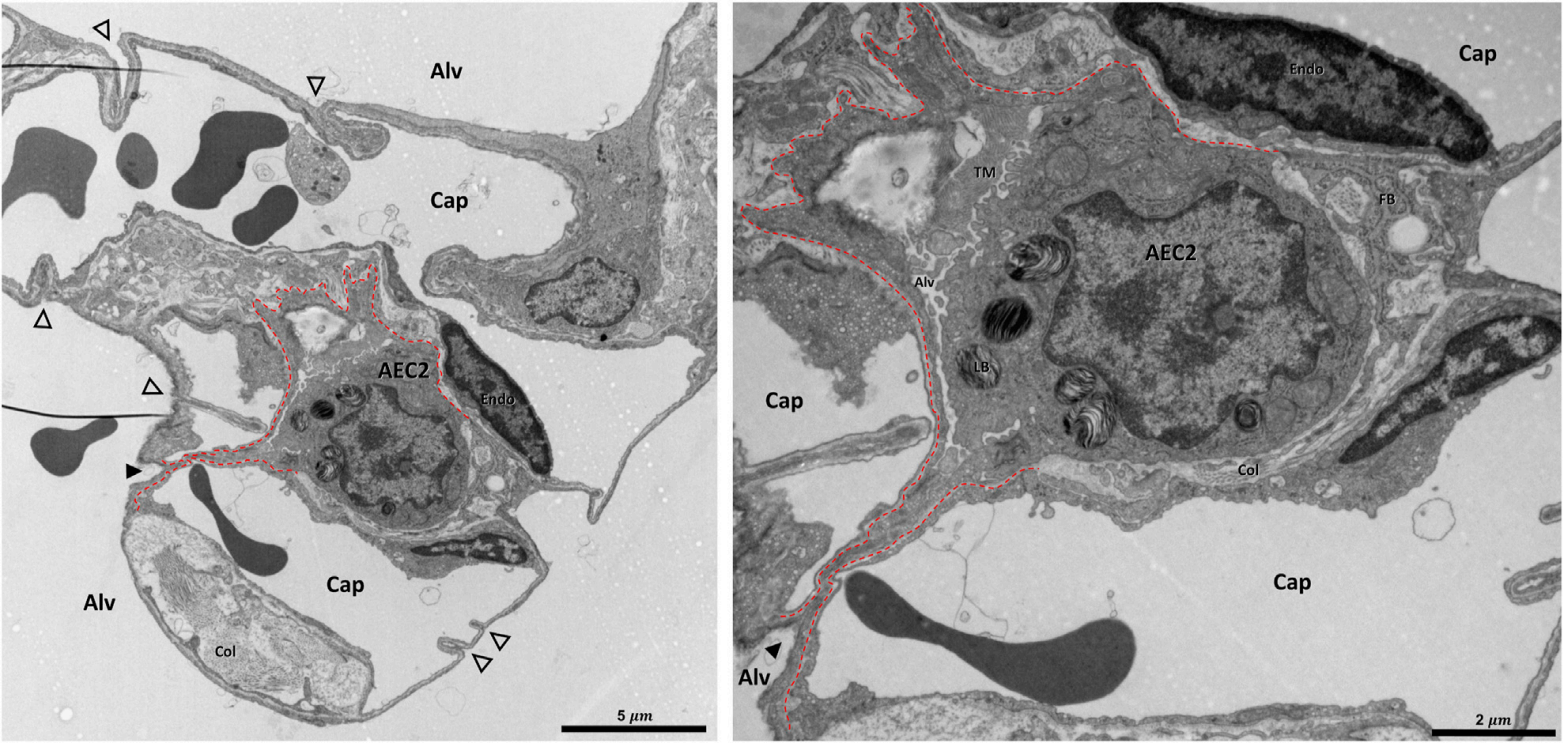
Pleat involving more components of the interalveolar septa: A healthy rat lung was fixed *in vivo* by vascular perfusion *via* the vena cava caudalis at an airway opening pressure of 5 cmH_2_O on expiration after two recruitment maneuvers (3 s pause at 30 cmH_2_O) (Knudsen et al., 2018). The alveolar airspaces (Alv) and the alveolar capillary network (Cap) is open but contains red blood cells. The capillaries are lined by endothelial cells (Endo). The filled arrowhead points at the entrance to a pleat, the black dashed line marks the run of the epithelial basement membrane into the pleat. The pleat is partly bordered by the apical plasma membrane of an alveolar epithelial type 2 cell (AEC2) with its characteristic organelle, the lamellar body (LB). Moreover, the pleat contains some fluid and intraalveolar surfactant, represented by tubular myelin (TM). Underneath the AEC2, interstitial tissue is located, e.g. collagen fibrils (Col) and fibrobasts (FB) are visible. Note that two dark lines at left of left image are an artifact due to folding of the ultrathin section, not part of the tissue structure. The empty arrowheads point at pleats formed exclusively by the blood-gas barrier.

**FIGURE 11:**
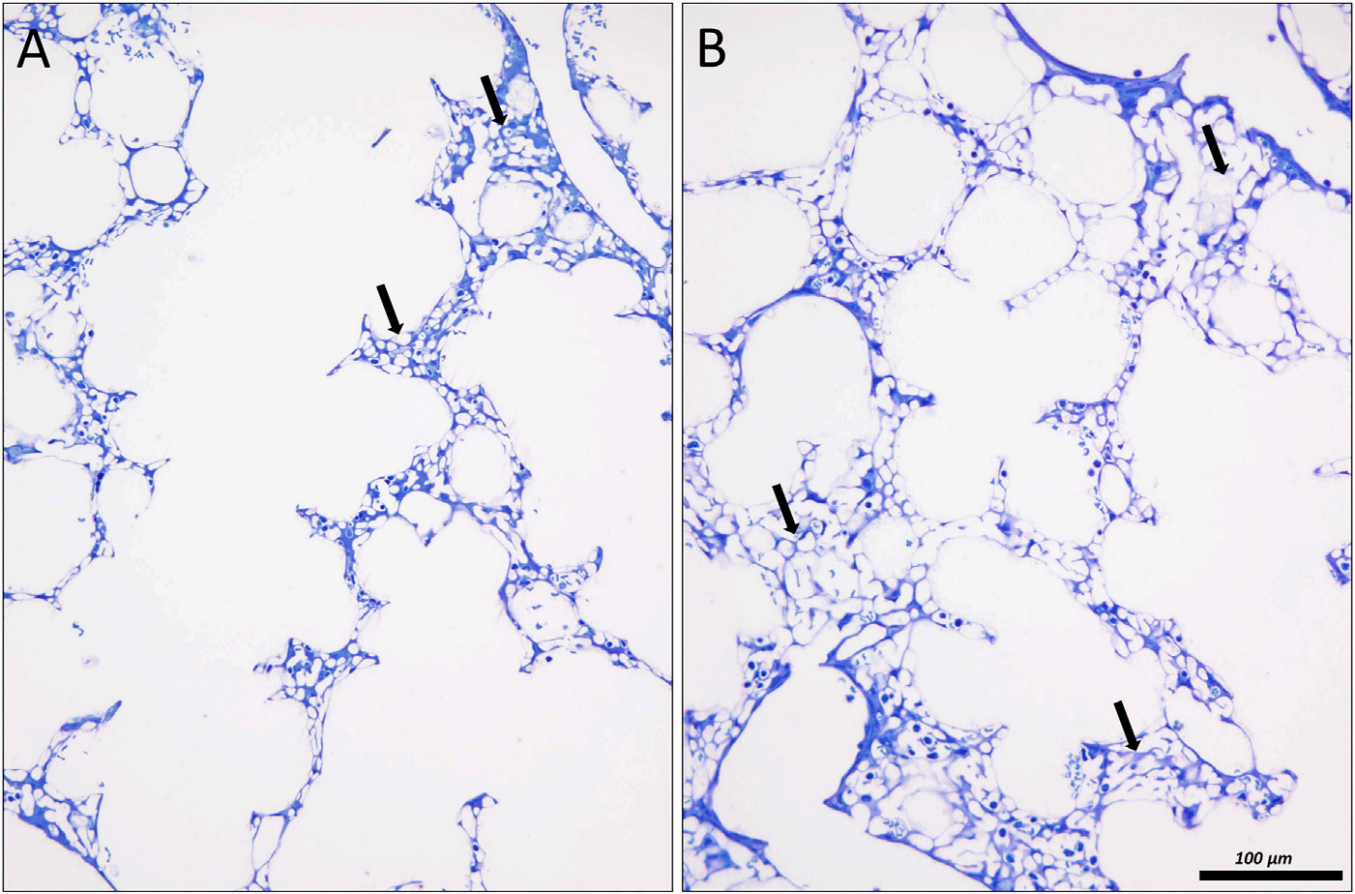
Lung injury and microatelectases: In **(A)** an image of the lung of a surfactant protein B knock out mouse is shown, fixed *in situ* by vascular perfusion *via* the right ventricle at an airway opening pressure of 10 cmH_2_O on expiration two recruitment maneuvers (3 s pause at 30 cmH_2_O) (Ruhl et al., 2019). The alveolar ducts are enlarged. Alveolar airspaces are rare and appear to be shallow. Instead, microatelectases (arrows) can be identified as seemingly thickened interalveolar septa characterized by a conglomeration of capillaries. In **(B)** similar findings can be observed in a rat lung 1 day after instillation of bleomycin to induce lung injury. The lung was fixed *in vivo* by vascular perfusion *via* the vena cava caudalis at an airway opening pressure of 10 cmH_2_O on inspiration, coming from 1 cmH_2_O after two recruitment maneuvers (3 s pause at 30 cmH_2_O).

Pleats are where parts of the interalveolar septal epithelial surface is not exposed to air. Instead, sections of the blood-gas barrier that form pleats are folded upon themselves, with a thin intervening layer of fluid as shown schematically in Figure 13. The detection of pleats requires electron microscopic resolution. Whether such pleats exist in healthy lungs *in vivo* during spontaneous breathing or mechanical ventilation is not entirely clear. Many studies performed under *ex vivo* as well as *in vivo* conditions describe the existence of pleats in lung tissue fixed by vascular perfusion (Gil and Weibel, 1972; Bachofen et al., 1987; Tschumperlin and Margulies, 1999; Knudsen et al., 2018). Oldmixon and Hoppin investigated lungs fixed *in vivo* at airway opening pressures between 0 and 45 cmH_2_O with varying volume history. Based on light and electron microscopic imaging they concluded that the occurrence of pleats is governed by the volume history and that pleats were not present as long as the airway opening pressure remained above 2-3 cmH_2_O (Oldmixon and Hoppin, 1991). Other investigators, however, noted that pleats were not rare events at the electron microscopic level even after recruiting the lungs and performing fixation at pressures from 1 to 25 cm H_2_O on expiration from TLC (Tschumperlin and Margulies, 1999; Knudsen et al., 2018). Pleats can either involve the blood-gas barrier exclusively or result from folding of the complete interalveolar septum. Pleats exclusively formed by the blood-gas barrier can be found in healthy rat lungs, fixed *in vivo* by vascular perfusion at end-inspiration and end-expiration, and form sickle-shaped invaginations into the underlying capillaries (Figures 8, 9). Pleats involving more than just the blood-gas barrier can predominantly be observed at septal junctions or between the meshwork of the alveolar capillary network (Figures 2, 3, 10). The septum piles up so that a multilayer of the alveolar capillary network is present in a seemingly thickened interalveolar septum. In the depth of such a pleat AE2 cells can often be encountered (Figures 2, 3, 10). Due to the arrangement of the alveolar capillary network in layers, these pleats may also become visible at light microscopic level. The presence of high surface tension is accompanied by more pronounced formation of pleats and piling up of complete septa thereby forming conglomerations of collapsed alveoli, also referred to as microatelectases (Figure 11). In general, the formation of pleats leads to a loss of air-exposed alveolar epithelial surface area.

#### 3.3.2 Recruitment and derecruitment of complete alveoli

In healthy lungs the mechanisms of recruitment and derecruitment of complete alveoli have been suggested from *ex vivo* experiments of isolated and perfused lungs where volumes were allowed to fall below the residual volume so that partial collapse was present before re-expansion and fixation (Gil and Weibel, 1972; Bachofen et al., 1987). Although this scenario is not generally physiologically relevant, it could describe the condition of a pneumothorax. *In vivo* microscopy of an initially partially collapsed lung provided evidence that alveolar recruitment (or the unfolding of septa forming the boundaries of a cluster of alveoli) is involved during the first respiratory cycles to adapt to lung volume changes. Computational simulations indicate that alveolar recruitment can explain the hysteresis of the first pressure-volume loops of a degassed lung (Bachofen et al., 1987; Carney et al., 1999; Bates and Irvin, 2002). Further imaging studies in healthy lungs using intravital microscopy, optical coherence tomography, and sections of fixed tissue where the lungs were kept above the residual volume do not provide evidence for alveolar recruitment or decruitment (Pavone et al., 2007; Perlman et al., 2011; Tabuchi et al., 2016; Knudsen et al., 2018; Ruhl et al., 2019; Smith et al., 2020). Hence, the healthy lung and the alveoli can be considered to be stable in presence of an intact surfactant system (Fung, 1975).

In contrast, severe abnormalities in alveolar dynamics including alveolar recruitment/derecruitment have been observed using intravital microscopy in lung injury induced, e.g., by lavage with detergents to remove surfactant or injurious mechanical ventilation (Schiller et al., 2001; Kollisch-Singule et al., 2020). However, other investigators did not find any evidence of intratidal recruitment/derecruitment during mechanical ventilation with physiological tidal volumes using similar imaging techniques (Mertens et al., 2009; Tabuchi et al., 2016; Nguyen and Perlman, 2018; Grune et al., 2019). Instead, these studies showed heterogeneous and asynchronous ventilation patterns in subpleural alveoli. These heterogeneous alveolar dynamics are characterized by subpopulations of alveoli showing decreased size changes while others are overdistended. Asynchronies include, e.g., inverse alveolar ventilation, alveolar stunning as well as the phenomenon of alveolar Pendelluft. The later results in a decrease in alveolar size during an inspiratory hold while other alveoli increase in size. Some of these abnormal patterns of alveolar dynamics can, at least in part, be explained by alveolar interdependence at the interface between injured and healthy lung regions. Alveolar injury can manifest as vascular leakage with alveolar edema accumulation or microatelectases with relatively minor alveolar fluid accumulation. Both of these alveolar-level injury manifestations exert tethering forces on the surrounding alveoli that affect their behavior during ventilation.

Derecruitment has been hypothesized to occur in the acutely injured lung when the transpulmonary pressure drops below a certain value termed alveolar closing pressure. Likewise, recruitment of alveoli during inspiration requires the transgression of an alveolar opening pressure that is greater than the closing pressure (Bates and Irvin, 2002). Using design-based stereology and injured lungs fixed at different airway opening pressures during expiration from TLC, several studies have explored how the distribution of alveolar closing pressures is related to the degree of injury using stereology (Knudsen et al., 2018; Ruhl et al., 2019; Smith et al., 2020). In healthy lungs, the number of alveoli per lung remained stable with airway opening pressures ranging from 20 down to 1 cmH_2_ *O. Minor* injury shortly after bleomycin challenge induced alveolar instability in a cohort of alveoli at airway opening pressures below 5 cmH_2_O. With injury progression the pressure needed to prevent the collapse of unstable alveoli was increased to 10 cmH_2_O and an additional cohort of alveoli was identified that could not be recruited with pressures up to 30 cmH_2_O. Furthermore, the alveolar opening pressures were much higher than the alveolar closing pressures so that during ventilation with physiological tidal volumes and PEEP <5 cmH_2_O hardly any intratidal alveolar recruitment/derecruitment could be detected (Knudsen et al., 2018). A VILI induced by high tidal volumes suggests three alveolar phenotypes after vascular perfusion fixation at different airway pressures: apparently healthy alveoli that were stable at low lung volumes, unstable but recruitable alveoli that collapsed at airway opening pressures below 5 cmH_2_O, and a third cohort that was flooded with edema and not recruitable (Smith et al., 2020). These findings transfer at least in part to clinical ARDS (Caironi et al., 2010; Cressoni et al., 2017).

Alveolar instability might also be of relevance in the pathophysiology of fibrosing lung diseases (Knudsen et al., 2017). In lungs of patients suffering from IPF, dysfunctional AE2 cells have been identified by many investigators (Parimon et al., 2020). In a recent study, fibrosis-induced increased proliferation of and Notch signaling in AE2 cells were linked with defective processing of hydrophobic SP-B and SP-C both being of high importance for biophysical properties of surfactant (Wasnick et al., 2023). These observations explain the reduced surface tension lowering properties of alveolar surfactant from IPF patients (Günther et al., 1999). Accordingly, evidence of alveolar instability could be derived from end-expiratory high-resolution CT in subpleural regions of the IPF lung and also from studies investigating the origin of inspiratory Velcro crackles in IPF (Vyshedskiy et al., 2009; Petroulia et al., 2018). Velcro crackles are likely to result from to explosive and energy-rich re-opening of alveolar airspaces. With disease progression, it has been suggested that alveoli remained collapsed throughout the entire respiratory cycle and trigger lung injury in the surrounding tissue *via* mechanical stress (Albert et al., 2019). Remnants of collapsed alveoli with their former entrances overgrown by epithelial cells have been found in fibrotic tissue in IPF. This phenomenon has been termed collapse induration and is a feature of IPF, idiopathic interstitial pneumonia, and COVID-19 (Katzenstein, 1985; Myers and Katzenstein, 1988; Ochs et al., 2021). Since alveolar surface area is considerably decreased in IPF it is plausible that alveolar collapse and collapse induration play important roles in the pathophysiology of IPF (Coxson et al., 1997).

**FIGURE 12:**
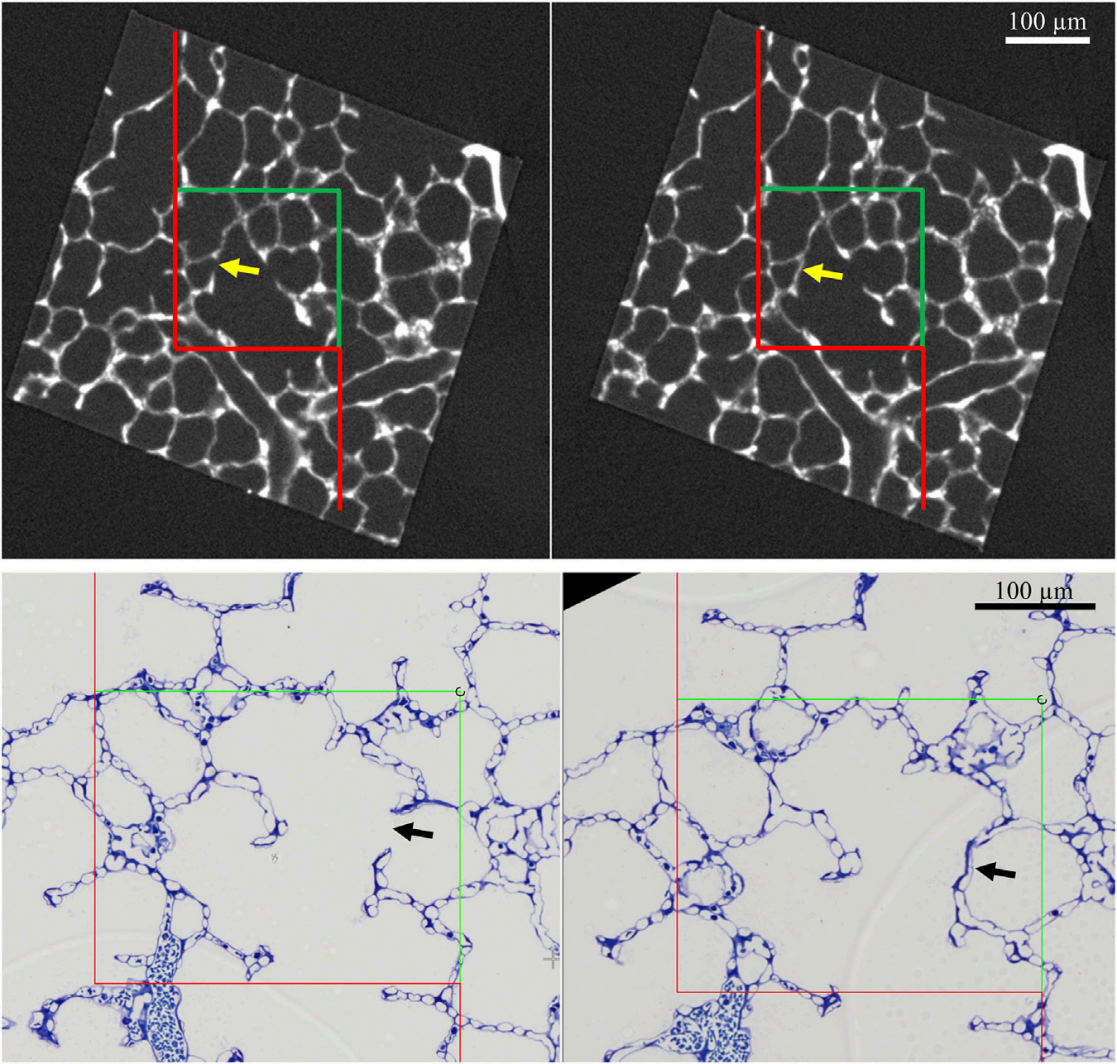
Counting open alveoli. Top: A mouse lung was fixed *in situ* by vascular perfusion *via* the right ventricle at an airway opening pressure of 10 cmH_2_O on expiration after two recruitment maneuvers (3 s pause at 30 cmH_2_O). Tissue was sampled, treated with OsO_4_ and embedded in epoxy resin (Ruhl et al., 2019). A tissue block was imaged by micro computed tomography (Nanotom M, Waygate Technology, Wunsdorf, Germany) at a voxel size 1 µm. A pair of images from the stack showing the same region is given. The distance from the top of the left image to the top of the right image is 4 µm. An unbiased counting frame of the area A_Frame_ is superimposed on the images. The counting frame area and the distance between the counting frames generates a test volume. In this test volume, the number of alveolar openings are determined as follows: alveoli open to the alveolar duct on one image but not on the other (arrow) are counted, provided that the opening to the duct is located within the counting frame and does not touch the red line (= forbidden line). Bottom: A healthy rat lung was fixed *in vivo* by vascular perfusion *via* the vena cava caudalis at an airway opening pressure of 10 cmH_2_O after two recruitment maneuvers (3 s pause at 30 cmH_2_O) (Knudsen et al., 2018). After randomized sampling and embedding, serial sections of a thickness of 1.5 µm were cut. The 1^st^ and the 4^th^ section of a consecutive row of sections was collected and randomized pairs of fields of view showing corresponding regions from these 2 sections were images. An example is given here. The arrow points at a counting event which is defined above.

The stereological parameters used to quantify the degree of alveolar derecruitment at different airway opening pressures included the alveolar number, the alveolar surface area, and the volume of the alveolar airspaces (Table 1). All these parameters can be determined both by light microscopy and with microcomputed tomography image stacks (Vasilescu et al., 2020). All parameters are calculated as absolute data per organ to avoid the reference trap. Of these three parameters, the alveolar number is the most technically challenging because it requires the use of a stereological test-volume. This can be generated by two microscopic sections from the same region separated by a known distance (= physical disector). Counting frames with a defined area are randomly superimposed on the microscopic sections (Ochs et al., 2004) so that the test volume is the product of the section separation distance and the area of the counting frame. Alveoli, that are open to the alveolar duct on the one section but not on the other, are counted if the counting event is within the counting frame (Figure 12). Scrolling through a microcomputed tomography image stack is a very efficient alternative to light microscopy when counting alveolar openings to determine alveolar number (McDonough et al., 2015; Knudsen et al., 2021). Another very efficient way to quantify microatelectases is the determination of the volume of collapsed and recruited septa by means of point-counting. Here, collapsed septa in adult lungs are defined by the existence of more than one layer of the alveolar capillary network, provided that the capillaries are open and can be identified accurately (Rizzo et al., 2022) (Figure 11).

**TABLE 1:** Design-based stereological parameters to study the micromechanics of interalveolar septa. Note, a forth mechanism is shape change due to mechanisms other than stretching/de-stretching or recruitment/de-recruitment (e.g. Reimelt et al., 2023). An example is the straightening of the curvy run of the blood-gas barrier which can occur independently from stretching or recruitment of the blood-gas barrier. As a result, the alveolar volume increases but the surface area of the air-exposed alveolar epithelium remains stable. * Did not determine surface area using stereological test-systems.

Mechanism	Imaging modalities	Stereological parameter	Comments	References
Alveolar recruitment/derecruitment (R/D)	Light microscopy, Micro computed tomography and synchrotron-based micro computed tomography	**First choices:**	High correlation with lung mechanical impairment	e.g. Ochs et al. (2004), Knudsen et al. (2018), Beike et al. (2019), Smith et al. (2020)
		Number of open alveoli per lung (Figure 12)		
		Volume of microatelectases/collapsed septa per lung (Figure 11)		e.g. Lutz et al. (2015), Ruwisch et al. (2020), Rizzo et al. (2022)
		**Second choices:**		e.g. Knudsen et al. (2018), Beike et al. (2019), Smith et al. (2020)
		Alveolar surface area per lung		
		Volume of alveolar airspaces per lung (Figure 7)	No proof of alveolar R/D	e.g. Lutz et al. (2015), Knudsen et al. (2018), Beike et al. (2019), Smith et al. (2020)
		Volume of interalveolar septa per lung and mean thickness of inter-alveolar septa (calculated from surface to volume ratio)	Mean thickness of interalveolar septa correlates inversely with number of open alveoli	e.g. Lutz et al. (2015)
Recruitment and derecruitment of pleats	Electron microscopy	Total surface area of alveolar epithelium hidden in pleats	In an air-filled lung the surface area of the alveoli does not equal the surface area of the alveolar epithelium	Bachofen et al. (1987), Ruhl et al. (2019), Engelmann et al. (2021)
		Total surface area of alveolar epithelium exposed to air		
		Total surface area of epithelial basement membrane (Figure 14)		
Stretching of the blood-gas barrier	Electron microscopy	Total surface area of epithelial basement membrane (Figure 14)	Changes in the surface area are directly linked with stretching/strain of epithelium and endothelium	Bachofen et al. (1987), Tschumperlin and Margulies (1999)*, Ruhl et al. (2019), Engelmann et al. (2021)

#### 3.3.3 Folding and unfolding of pleats without recruitment and derecruitment of complete alveoli

Septal pleats invaginating the blood-gas barrier or the entire interalveolar septa, without the derecruitment of complete alveoli, have been described based on electron microscopy in healthy lungs from mice and rats (Tschumperlin and Margulies, 1999; Knudsen et al., 2018; Ruhl et al., 2019). These pleats hide alveolar epithelial surface area and are typically filled with a thin layer of protein-containing liquid. Intraalveolar surfactant, e.g. tubular myelin can often be seen at the entrance to the pleats. These pleats, and the associated liquid layer, have been investigated using design-based stereology at the electron microscopic level at different airway pressures (Ruhl et al., 2019). Decreasing the airway opening pressure to 2 cmH_2_O on the expiratory limb of the pressure volume loop increased the frequency of observed pleats in healthy mice and rats, a finding that correlates with an increase in tissue elastance (Knudsen et al., 2018). The surface area of alveolar epithelial cells covered by the intra-pleat fluid increased while the aerated surface area decreased. At higher lung volumes, the majority of protein containing fluid was located in the corners of alveoli. With decreasing lung volume, liquid was integrated into the increasing number of pleats and formed a very thin layer so that the mean thickness of the liquid layer determined via stereology decreased. From these observations it is likely that there is recruitment and derecruitment of pleats accompanied by a reorganization of the alveolar lining fluid, at least under quasi-static conditions at lung volumes within the range of physiological breathing. From a physiological point of view this is an appealing mechanism since it allows the alveolar volume and surface area to change without strain of the blood-gas barrier. In other words, with inflation the aerated alveolar surface area increases while the thickness of the blood-gas barrier and surface area of the epithelial BM remain stable. Septal stretching would result in an increase of the surface area of the epithelial BM combined with thinning of the blood-gas barrier (Bachofen et al., 1987; Leuenberger et al., 2012). Lung physiological studies in humans provide indirect evidence of the existence of this micromechanical mechanism under *in vivo* conditions. A modeling-based approach indicates that the measured diffusion capacity with increasing lung volume of up to 80% TLC could best be explained by an increase in air-exposed alveolar surface area without a decrease in the diffusion membrane thickness, suggesting that recruitment of pleats occurs up to 80% of TLC (Miserocchi et al., 2008).

In an *ex vivo* lung study, Bachofen and co-workers used design-based stereology to quantify the alveolar surface area (S_A_) and the surface area of the alveolar epithelial BM (S_ebl_) at quasi-static conditions during pressure volumes loops starting with a pressure near zero (Bachofen et al., 1987). The S_A_-to-S_ebl_ ratio as well as the S_ebl_ increased during inflation, indicating that the increase in surface area is a result of both stretching of the blood-gas barrier and recruitment of surface area hidden in pleats. However, these findings may not be directly applicable to the *in vivo* situation since inflation started below residual volume. Furthermore, Bachofen et al. did not differentiate between recruitment of whole alveoli and recruitment of pleats of the interalveolar septa of those alveoli which are already open. In a later study, lungs were fixed *in situ* at either 10 or 2 cmH_2_O following deflation from 30 cmH_2_O, thus maintaining a volume history above the residual volume (Ruhl et al., 2019). There was no significant difference in alveolar number, indicating that whole-alveolar derecruitment did not occur. The S_A_-to-S_ebl_ ratio decreased from 0.75 to 0.52 during expiration, supporting the conclusion that the loss of aerated alveolar epithelial surface area during deflation is due to both de-recruitment of pleats and de-stretching of the blood-gas barrier because derecruitment without de-stretching would have resulted in a decline of the ratio from 0.75 to 0.42. Estimation of the differential contribution of these two mechanisms to the decline in aerated alveolar surface assigns approximately 40% to the formation of pleats and derecruitment and 60% to de-stretching (Ruhl et al., 2019). However, these studies do not reveal the precise range of lung pressures and volumes during inflation and deflation where these processes occur, and whether they occur during dynamic breathing.

**FIGURE 13:**
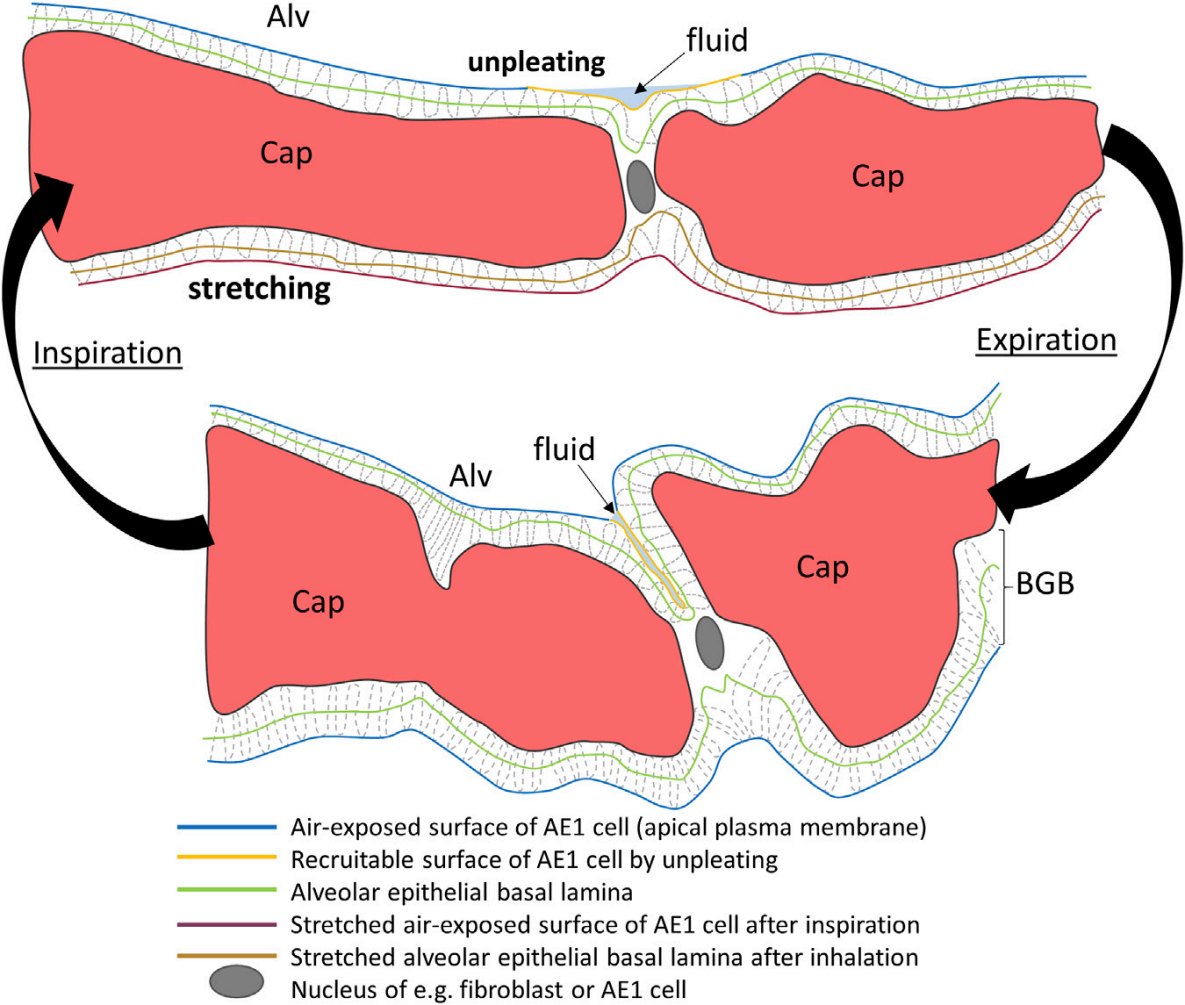
Micromechanics of the blood-gas barrier. Schematic of an interalveolar septum at end-expiration (bottom) and end-inspiration (top). The end-expiratory drawing is based on an electron microscopic image and shows in the upper blood-gas barrier (BGB) a pleat filled with fluid (light blue). The surface area of the involved alveolar epithelial cell is hidden in the pleat (yellow) and not exposed to air. After inspiration, the pleat has been opened and its surface area, although still covered by fluid is now exposed to the alveolar lumen. As a result, there is an increase in surface area and adaptation to changing alveolar size without stretching of the alveolar epithelial cell or the epithelial basement membrane (green). The BGB at the bottom of the septum however, does not have a pleat. The BGB is therefore stretched during inspiration which results in an increase in the surface area of both air-exposed apical membranes of alveolar epithelial cells and epithelial basement membrane. The BGB is thinned due to stretching. Capillaries (Cap) and the BGB are also subject to shape changes to adapt to an increase in alveolar volume during inspiration which do not result in an increase in the surface area of the basement membrane or apical plasma membrane of epithelial cells. This schematic is based on classical transmission electron microscopic images which are not able to visualize the complete liquid lining layer. Please note, that the fixation process with glutaraldehyde is based on cross-linking of proteins. Hence, only those parts of the liquid lining layer which contain proteins can be visualized and are given in this schematic.

As mentioned earlier, the recruitment of pleats is a way to adapt to volume changes without stretch of the blood-gas barrier but it is also associated with a reorganization of the liquid lining layer and associated pulmonary surfactant. The opposing blood-gas barriers of the pleats are peeled off each other during inflation and the fluid oscillates on the surface of the epithelial cells (Figure 13). Based on *in vitro* experiments and computational modeling this opening process can be linked with potentially harmful forces acting on the epithelial lining if surface tension is elevated due to surfactant dysfunction, a typical feature of lung injury (Bilek et al., 2003; Kay et al., 2004; Naire and Jensen, 2005; Ravasio et al., 2011; Hobi et al., 2012). Hence, the recruitment process of pleats might be detrimental in conditions of high surface tension by causing additional injury of the blood-gas barrier and dysfunction of the AE2 cells, a mechanism referred to as microatelectrauma.

#### 3.3.4 Stretching of the blood-gas barrier

**FIGURE 14:**
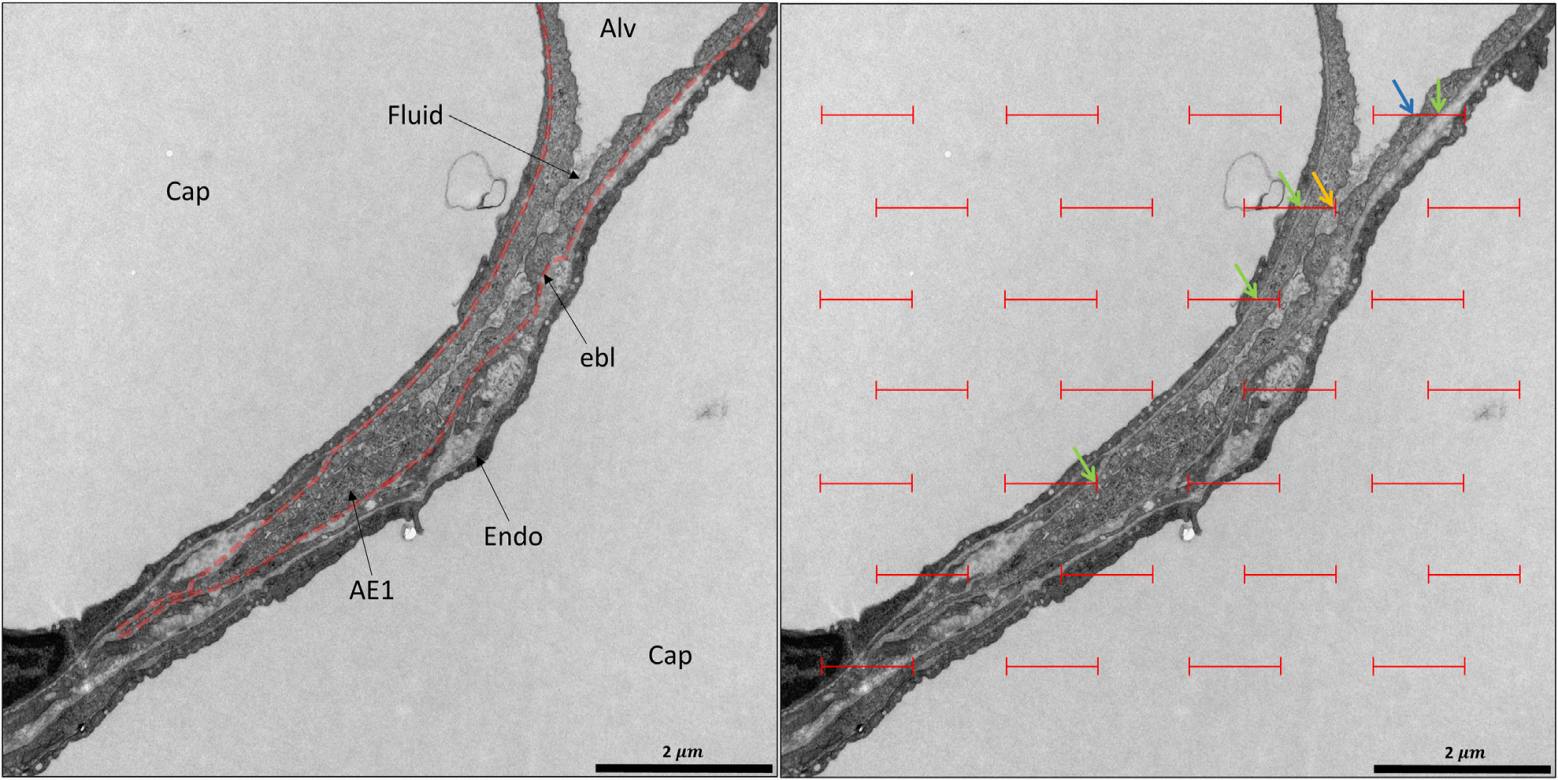
Design-based stereology of the blood-gas barrier: A healthy rat lung was fixed *in vivo* by vascular perfusion *via* the vena cava caudalis at an airway opening pressure of 10 cmH_2_O after two recruitment maneuvers (3 s pause at 30 cmH_2_O) (Knudsen et al., 2018). In the center, a pleat filled with protein-containing fluid and bordered by the blood-gas barriers can be seen. On the left, the epithelial basement membrane (ebl) is delineated (dashed red line). While at the top right corner, the alveolar epithelium is exposed to air (Alv), the pleat hides epithelial surface area which might be recruitable on inspiration. The pleat is filled by a grayish material representing preserved parts of the liquid-lining layer. In order to quantify the surface area covered by air or hidden within pleats, test lines can be projected on randomized electron microscopic images for intersection counting as shown on the right. Line segments of a certain length are superimposed on the image. The probability of these line segments to intersect air covered or hidden alveolar epithelium is proportional to the collective length (L) of the line segments but also to the surface density of, e.g. air-covered or hidden alveolar epithelium. Hence, intersection (I) counting can be applied to determine the surface density (Sv) of the desired structures, given by the equation Sv = 2*I/L. Intersections are indicated as follows: blue arrow: air-covered alveolar epithelial surface area; yellow arrow: hidden alveolar epithelial surface area. The same line segments can be used to determine the surface area of the epithelial basement membrane, a parameter eligible to quantify stretch of the blood-gas barrier. The green arrows point at intersections of the line segments with the epithelial basement membrane.

A few studies have quantified the S_ebl_ under quasi-static conditions, using design-based stereology or comparable unbiased methods, to reveal the degree of stretching/de-stretching and pleating/unpleating of the blood-gas barrier as lung volume changes (Figure 14). These studies intended to define the range of lung volumes in which stretching/de-stretching occurs (Bachofen et al., 1987; Tschumperlin and Margulies, 1999). The investigated species and volume histories differed between studies; thus, direct comparison is difficult. As mentioned earlier, Bachofen et al. started inflation of isolated and perfused rabbit lungs with transpulmonary pressures close to zero and fixed the lungs during the first and second respiratory cycle. The fixed lungs covered the range of lung volumes between 40% (≈ functional residual capacity) and 100% of TLC during both inspiration and expiration. In the second respiratory cycle S_ebl_ increases by 26% at a lung volume of 80% TLC compared to 40% TLC. Tschumperlin and Margulies ventilated explanted rat lungs for several respiratory cycles with transpulmonary pressures well above the residual volume to avoid any lung collapse. Lungs were fixed at 100% TLC and during expiration at lung volumes corresponding to 82%, 60%, 42% and finally 24% of TLC which occurred at transpulmonary pressures from 25 to 2 cmH_2_O. At TLC S_ebl_ was 40% larger compared to a lung volume of 24% TLC. However, the majority (approximately 70%) of the changes in S_ebl_ could be documented between 100% and 80% TLC. Hence, the authors concluded that stretching/de-stretching of the blood-gas barrier predominantly takes place at lung volumes above 80% TLC and thus above the range of lung volume in which quiet spontaneous breathing takes place. Computational modeling using measurements of pulmonary diffusion capacity at 80% and 100% TLC in humans supports these findings. The observed increase in diffusion capacity could best be explained by a thinning of the blood-gas barrier and thus stretching instead of increase in surface area by unfolding processes (Miserocchi et al., 2008). Rühl et al. also quantified the S_ebl_ during expiration in mouse lungs. Because the lung fixation was carried out at set transpulmonary pressures of 2 and 10 cm H_2_O, instead of lung volume, a direct comparison to the other publications is difficult. The two-dimensional strain of the blood-gas barrier occurring between those pressures (calculated from S_ebl_) was 23%, a value a bit larger than that one calculated by Tschumperlin and Margulies between 24% TLC (transpulmonary pressure = 2 cmH_2_0) and 82% TLC (transpulmonary pressure = 8.8 cmH_2_O) which was in the range of 12%–16%. The current available data suggest that within the range of physiological lung volumes both unfolding/folding and stretching/de-stretching occur in parallel as illustrated in Figure 13.

The strain of the blood-gas barrier is not homogenous, and the regional deformation changes with lung volume. This strain heterogeneity was investigated using design-based stereology and electron microscopy to estimate the surface area of the epithelial BM covered by AE1 and AE2 cells in mice at 10 and 2 cmH_2_O on expiration. While the surface area of the BM covered by AE2 cells remained rather stable, the area covered by AE1 cells differed between those two pressures (Ruhl et al., 2019). These observations are supported by confocal microscopy of lungs inflated to different lung volumes (Perlman and Bhattacharya, 2007). Thus, in the range of lung volumes of physiological breathing the strain of AE1 cells is larger than that one of AE2 cells. This may occur because at lower lung volumes the AE2 cells are preferentially located within pleats and those pleats must be unfolded before the AE2 cells are subjects to strain. Based on these considerations it appears to be reasonable that AE2 cells are only stretched at higher lung volumes, e.g., above 80% of TLC.

## 3 Conclusion

Recent innovations in three and four-dimensional imaging techniques have expanded our knowledge of the micromechanics of acini and alveoli. At the largest scale, these dynamics are manifest as volumetric strain which can be quantified using imaging techniques with light microscopic resolution. However, the micromechanical mechanisms by which the interalveolar septa adapt to alveolar volume changes during breathing or mechanical ventilation, including tissue strain and recruitment of septal pleats, require inclusion of electron microscopic resolution. Pleats can involve either the blood-gas barrier alone, creating invaginations into the alveolar capillaries, or small folds of the entire interalveolar septum. In the latter case, the folding predominantly occurs between the piles of the alveolar capillary network located at the junctions of inter-alveolar septa (the corners of the alveoli). Pleating is a consequence of a complex interplay of tissue tension (fiber system) and surface forces at the air-liquid interface and result in a loss of alveolar surface area. In the context of lung injury, surface tension can increase considerably so that complete alveoli collapse and form microatelectases. These induce potentially harmful stresses on adjoining interalveolar septa, a mechanism discussed to be involved in ventilation-induced lung injury but also the progression of fibrosis lung diseases. The micromechanical mechanisms of alveolar septal deformation can be quantified using design-based stereology and transmission electron microscopy. Data suggest that in healthy lungs, with a volume history above the residual volume, recruitment of entire alveoli is infrequent. Instead, volume changes up to 80% of TLC are accommodated by recruitment of septal pleats and septal stretching which, in combination, yield increased gas exchanging surface area (Figure 13). However, above 80% TLC, stretching might dominate recruitment.
